# TRP-Net: A Text-Guided Dual-Modal Network for Potato Leaf Lesion Detection Under Complex Imaging Conditions

**DOI:** 10.3390/plants15193015

**Published:** 2026-09-30

**Authors:** Yiqing Zeng, Jiale Gu, Xiaomei Yu, Yuanhan Luo, Peng Wang, Mingfang He

**Affiliations:** Artificial Intelligence Application Research Institute, Central South University of Forestry and Technology, Changsha 410004, China; 20232170@csuft.edu.cn (Y.Z.); 20242140@csuft.edu.cn (J.G.); t20051927@csuft.edu.cn (X.Y.); t20061888@csuft.edu.cn (Y.L.); t20162306@csuft.edu.cn (M.H.)

**Keywords:** potato leaf disease, early blight, late blight, text-guided fusion, counterfactual intervention, residual cross-modal attention, TRP-Net, object detection

## Abstract

Potato leaf diseases seriously affect potato yield and quality. Accurate recognition and localization of visible lesions associated with potato early blight and late blight can support preliminary disease screening and targeted field inspection. Object detection can identify disease categories and locate lesion regions, providing reliable information for disease management. However, potato leaf diseases usually occur in complex field environments, where illumination changes, shadows, color shifts, leaf texture interference, and imaging blur may weaken lesion feature representation. In addition, early blight and late blight lesions often have small scales, weak boundaries, irregular shapes, and scattered distributions, making detection prone to background misclassification, missed small lesions, and localization deviation. To address these problems, this study proposes TRP-Net, a text-guided dual-modal detection network, for the robust recognition and localization of visible lesions associated with potato early blight and late blight under complex imaging conditions. First, a Text-Anchored Counterfactual Intervention Module (TACIM) is proposed to construct environmentally perturbed images and constrain feature consistency, improving the robustness of disease representations. Second, a Residual Cross-Modal Attention module (RCMA) is introduced to guide visual features with textual semantics and enhance lesion boundaries and fine-grained textures. Third, a P4 Gaussian Position Distribution Auxiliary Loss (PGDAL) is designed to provide position distribution supervision during early training, improving small-lesion localization. Experimental results on the independent test set show that TRP-Net achieves 78.3% Precision, 73.7% Recall, 75.9% F1 score, 80.5% mAP50, and 34.6% mAP50–95. The detector-side network contains 2.79 M parameters and requires 5.2 GFLOPs. The performance gap between mAP50 and mAP50–95 indicates that precise lesion localization under stricter IoU criteria remains challenging.

## 1. Introduction

Potato is an important tuber crop widely cultivated worldwide. It is used as a staple food, vegetable, and raw material for food processing. Therefore, potato production contributes to food security, dietary diversity, and the development of agricultural processing industries [1,2,3]. During potato growth, leaf health directly affects the effective photosynthetic area of the canopy. Early blight and late blight are two major foliar diseases that can severely damage potato plants. Early blight is typically characterized by brown necrotic lesions with concentric rings, whereas late blight often produces irregular, dark-brown, or water-soaked lesions. As the lesions expand, infected leaves may develop chlorosis, necrosis, and premature senescence, resulting in a reduction in effective photosynthetic area. In severe cases, the diseases may also affect stems and tubers, ultimately reducing crop yield and quality.

Field surveys of potato diseases currently rely mainly on visual inspection by growers and agricultural technicians, whereas final disease confirmation usually requires professional assessment or laboratory testing. Visual inspection can be affected by examiner experience, illumination conditions, complex backgrounds, and visual fatigue. Laboratory tests provide more reliable confirmation, but their time, equipment, and personnel requirements limit their use for large-scale preliminary field screening [4,5]. Therefore, RGB images can be used to identify visible symptoms associated with early blight and late blight and to locate suspected lesions. This approach can support abnormal-leaf screening, targeted field inspection, subsequent sampling, and expert review, thereby improving the coverage and consistency of field surveys. The proposed method is limited to recognizing and localizing visible lesion patterns associated with early blight and late blight in RGB images. It is not intended to identify all potato diseases, detect latent infections before visible symptoms appear, or independently determine the causal pathogen without professional assessment or laboratory confirmation.

With the development of computer vision, image-based identification of potato foliar diseases has gradually shifted from manual observation to automated computational methods [6,7]. Early approaches typically used manually designed features based on lesion color, texture, shape, and area ratio, followed by classifiers such as support vector machines, random forests, or shallow neural networks. For example, Hylmi et al. [8] extracted RGB color histograms, gray-level co-occurrence matrix (GLCM) texture features, and lesion contours from potato leaf images and used a multiclass support vector machine with a linear kernel for disease classification. However, these methods rely heavily on manually selected features. Predefined color and texture descriptors cannot fully represent variations in disease symptoms, which limits model generalization [9].

These limitations have encouraged the transition from traditional machine-learning methods to deep neural networks for potato foliar disease analysis. By learning hierarchical representations directly from images, deep-learning methods reduce their dependence on manually designed features and have been applied to disease classification, lesion localization, and severity assessment [10,11]. For example, Sinamenye et al. [12] combined a convolutional neural network with a vision transformer for potato leaf disease classification. However, whole-image classification methods mainly provide image-level disease labels and generally cannot localize individual lesions or characterize their spatial extent. This limitation motivates the use of object detection for simultaneous disease recognition and lesion localization.

In deep-learning-based disease analysis, classification, image segmentation, and object detection produce different outputs. Classification networks assign a disease label to the entire image but do not identify lesion locations. Semantic segmentation provides pixel-level lesion regions but requires costly mask annotations and usually involves greater training and computational demands. Object detection simultaneously predicts disease classes and lesion bounding boxes, offering a practical balance among localization accuracy, annotation cost, and inference efficiency. Two-stage detectors, represented by Faster R-CNN, generally provide strong region proposal analysis but use relatively complex computational pipelines. In contrast, one-stage detectors, such as the YOLO series, directly predict class labels and bounding boxes. Their faster inference and flexible deployment make them more suitable for disease screening tasks involving large numbers of leaf images [13].

**Figure 1:**
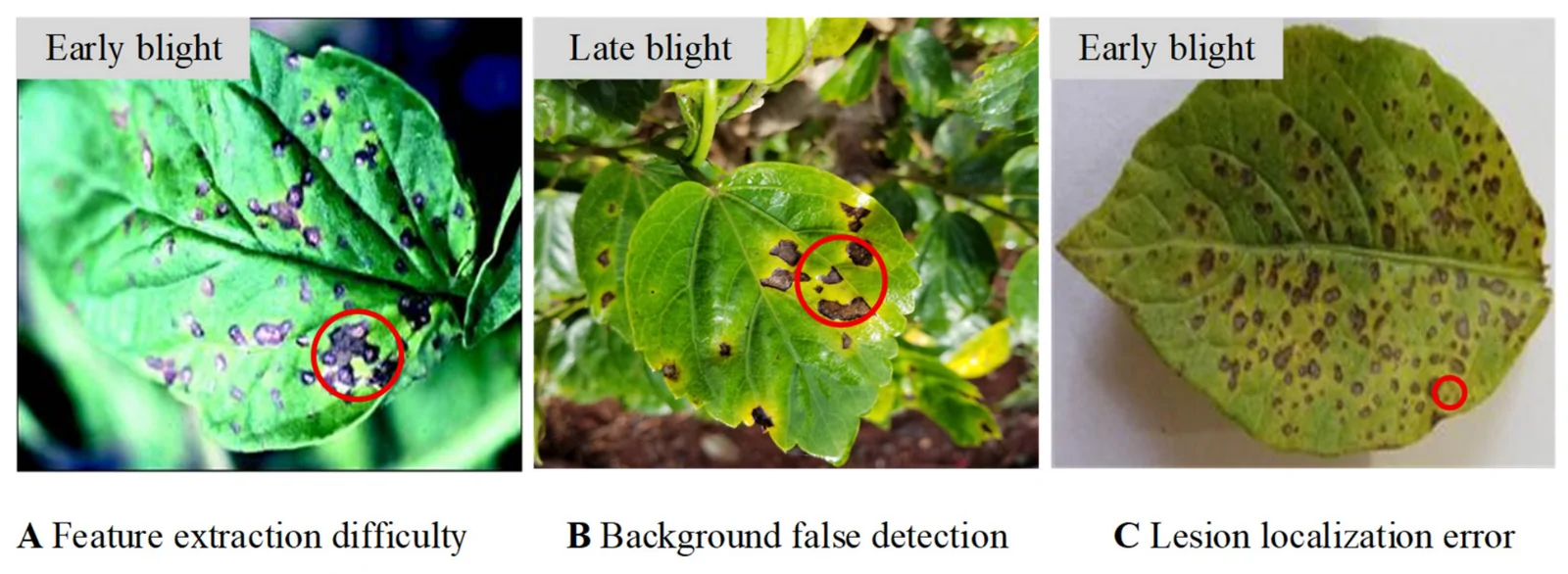
Problems with potato leaf disease detection. (**A**) Feature-extraction difficulty associated with environmental appearance variation in an early-blight image; (**B**) background interference from lesion-like leaf textures in a late-blight image; and (**C**) localization difficulty for small early-blight lesions. Red circles indicate representative regions illustrating the corresponding detection challenge. The images are illustrative examples from the potato dataset and do not represent model predictions.

As illustrated in Figure 1, the detection of potato early blight and late blight lesions still faces three interrelated technical challenges:

(a) Environmental variation causes unstable feature extraction. Field images are easily affected by illumination, shadows, color shifts, and blur, causing lesions of the same disease to appear differently and increasing the difficulty of feature extraction.
(b) Leaf textures cause background misclassification. Normal leaf features, such as veins, folds, and shadows, may resemble lesion patterns, leading detection networks to misclassify background regions as diseased areas.
(c) Complex lesion distributions make localization difficult. Potato lesions vary considerably in size, number, and position and often overlap or merge, which can cause bounding-box shifts, missed small lesions, and inaccurate localization.

To address unstable feature extraction caused by environmental variation in field images, previous studies have mainly used image enhancement, data augmentation, and robust feature learning. Ye et al. [14] applied an improved Enlighten GAN to enhance low-light tea disease images. Tang et al. [15] combined multiscale convolution, attention mechanisms, and adjacent-layer feature aggregation to improve disease detection in complex backgrounds. Although these methods enhance image quality or visual feature representation, they mainly process each image independently. They do not directly constrain the consistency of lesion features under changes in brightness, shadows, color, blur, or noise [16].

To reduce false detections caused by visually similar background patterns, previous studies have mainly used channel and spatial attention mechanisms. For example, Liu et al. [17] proposed HACFM, which combines local and global attention to fuse multiscale features. However, these methods rely mainly on visual correlations within the image and may remain sensitive to lesion-like responses from leaf veins, shadows, folds, and senescent tissues [18]. Visual attention alone may therefore be insufficient to distinguish true lesions from structurally similar background regions, highlighting the need for complementary semantic guidance.

To address localization difficulties caused by complex lesion distributions, previous studies have mainly focused on bounding-box regression, sample assignment, and multiscale feature fusion. For example, Abulizi et al. [19] introduced MPDIoU to improve the localization of overlapping lesions, while ranking-based assignment strategies have been used to optimize positive–negative sample matching for small-object detection. However, these methods mainly improve box regression or sample assignment and do not directly preserve small-lesion responses or spatial information during successive downsampling [20]. This limitation highlights the need for auxiliary position supervision for small lesions.

Recent studies have increasingly explored multimodal and vision–language learning for plant disease analysis. Liu et al. [21] generated hierarchical disease descriptions using a vision–language model and fused visual and textual features through cross-attention for crop disease recognition. Xiong et al. [22] investigated automated disease-description generation with large multimodal models, including LLaVA, and multimodal fusion for crop disease recognition. Wu et al. [23] proposed PlantIF to model shared and modality-specific visual–text semantics for plant disease diagnosis. Beyond image-level recognition, Li et al. [24] developed PDC-VLD, an open-vocabulary multimodal detector that aligns image regions with textual representations for tomato leaf disease detection. These studies demonstrate the potential of textual semantics across different plant disease tasks. However, the use of image-level semantic guidance together with environmental consistency learning and position-aware supervision for fine-grained lesion detection remains insufficiently investigated.

Accordingly, this study examines whether image-level textual semantics and the three task-specific components of TRP-Net provide distinct benefits for potato lesion detection. We hypothesize that textual semantics complement visual features, TACIM improves representation stability under environmental variation, RCMA enhances discrimination between lesions and visually similar backgrounds, and PGDAL improves small-lesion localization through position-aware auxiliary supervision.

The main contributions of this study are summarized as follows:

1. This study constructed a finely annotated potato leaf disease dataset containing late blight and early blight, in which lesion regions were labeled with category information and bounding boxes to support disease recognition and localization.
2. TRP-Net, a vision–language detection network, was developed for visible potato lesion detection. The network was designed to address unstable feature extraction under environmental variation, interference from similar background patterns, and difficulties in localizing small lesions:
  (a) A text-anchored counterfactual intervention module (TACIM) was introduced. It generates environmentally perturbed images by varying brightness, color, shadows, blur, and noise. Feature consistency between the original and perturbed images is then constrained to reduce lesion feature shifts caused by changes in imaging conditions.
  (b) A text-guided boundary-enhanced attention module (RCMA) was proposed. Auxiliary semantic information describing lesion color, morphology, texture, and distribution guides lesion-region modeling. The module also strengthens lesion boundary and fine-grained texture responses to reduce interference from similar visual patterns, such as leaf veins and shadows.
  (c) A Gaussian position distribution auxiliary loss strategy (PGDAL) was introduced. During training, it provides intermediate features with supervision based on lesion position distributions. This strengthens spatial responses around the centers and neighboring regions of small lesions, reducing missed detections and localization errors caused by feature downsampling.
3. TRP-Net provides a text-guided framework for recognizing and localizing visible early blight and late blight lesions while maintaining low detector-side model complexity. Its outputs can support preliminary symptom screening, targeted field inspection, subsequent sampling, and expert assessment.

## 2. Results

This section evaluates the effectiveness of TRP-Net through a series of systematic experiments. First, the contribution of the TextGate pathway and the conditional effects of TACIM, RCMA, and PGDAL are examined on the validation set. Next, repeated factorial ablation experiments using five prespecified random seeds are conducted to evaluate the variability of the proposed configurations. Class-specific AP results, precision–recall curves, and the normalized confusion matrix are then analyzed to provide a more detailed evaluation of disease recognition and lesion localization. Subsequently, TRP-Net is compared with representative object detectors on the independent test set. Finally, an architecture transferability experiment is conducted on a tomato leaf disease dataset to evaluate the transferability of the proposed architecture.

### 2.1. Module Effectiveness Experiments

The individual effects of TACIM, RCMA, and PGDAL were evaluated in the following experiments.

#### 2.1.1. Effectiveness Experiment of TACIM

**Figure 2:**
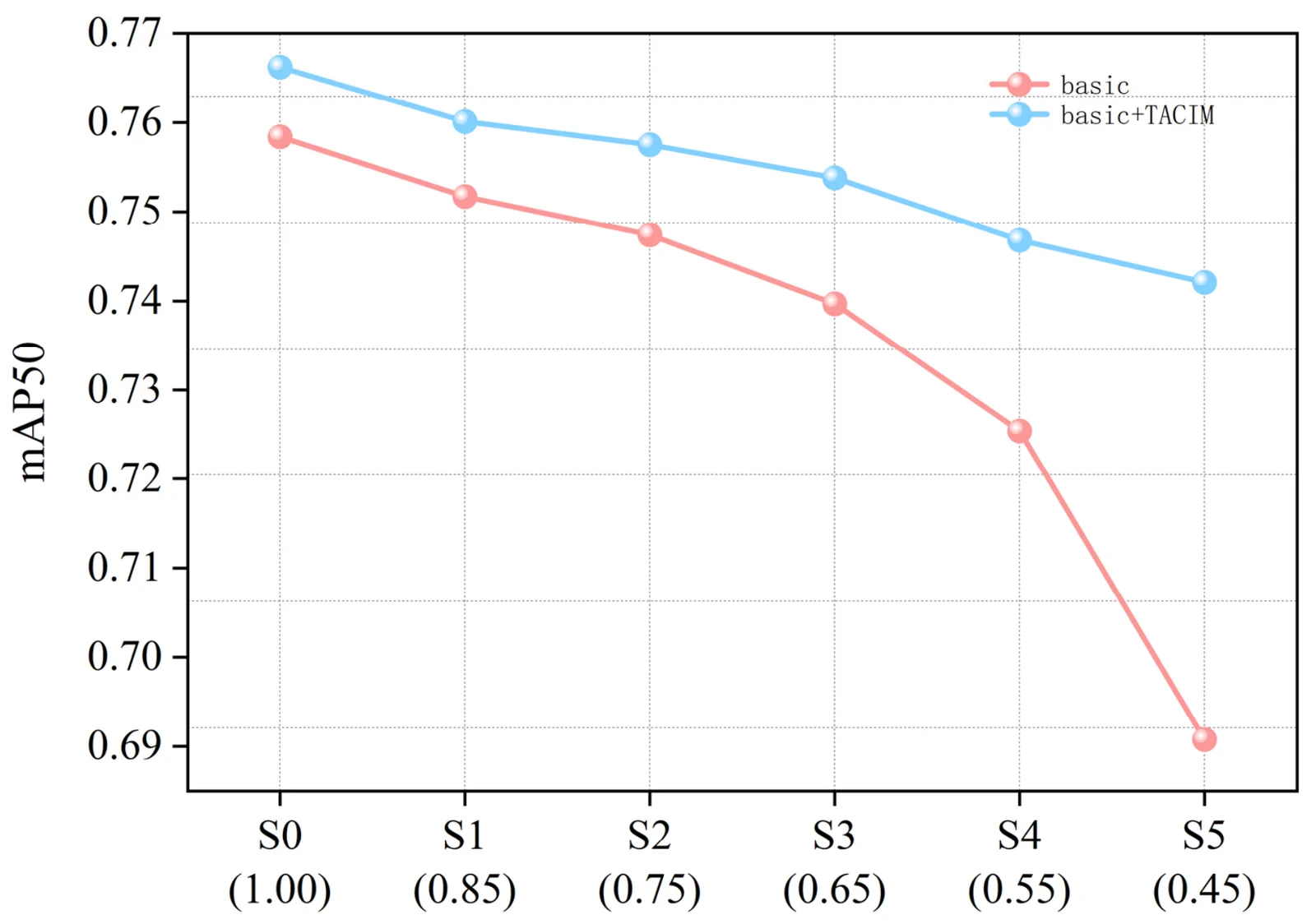
Low-light robustness evaluation on the potato validation set. The *x*-axis shows the six brightness conditions S0–S5, corresponding to brightness-scaling factors of 1.00, 0.85, 0.75, 0.65, 0.55, and 0.45, respectively; the *y*-axis shows mAP50 (%), calculated at an IoU threshold of 0.50. Red circles represent Basic, and blue squares represent Basic + TACIM. The same trained models and lesion annotations were used across all illumination conditions, with only image brightness modified. mAP50 was calculated from the complete precision–recall curve and therefore does not depend on a single fixed confidence threshold.

To assess the adaptability of TACIM to brightness variation, artificial low-light evaluation sets were constructed from the original validation set. Only image brightness was reduced, while image content, lesion classes, and bounding-box annotations remained unchanged. S0 denotes the original brightness condition and does not refer to random seed 0, whereas S1–S5 correspond to brightness coefficients of 0.85, 0.75, 0.65, 0.55, and 0.45, respectively. Basic and Basic + TACIM were evaluated under all six conditions using the same input size, confidence threshold, and post-processing settings. The corresponding mAP50 results are summarized in Table 1 and Figure 2. Representative lesion annotations are shown in Figure 2.

As the brightness coefficient decreased, both models showed a progressive decline in detection performance. Basic decreased from 75.84% mAP50 at S0 to 69.08% at S5, whereas Basic + TACIM decreased from 76.62% to 74.21%. The difference between the two models widened with increasing brightness reduction, from 0.78 percentage points at S0 to 5.13 percentage points at S5. Moreover, under the strongest perturbation condition, Basic and Basic + TACIM retained 91.09% and 96.85% of their respective S0 performance. Overall, the low-light evaluation shows a more gradual performance degradation for Basic + TACIM across the tested brightness levels.

#### 2.1.2. Effectiveness Experiment of RCMA

To evaluate the effects of different feature-enhancement modules, GSPN, ODA, FDAM, and RCMA were separately incorporated into the baseline model for comparison [25,26,27]. As shown in Table 2, RCMA achieved the highest mean Precision, Recall, F1 score, and mAP50 among the evaluated modules, reaching 75.61 ± 1.32%, 72.43 ± 0.80%, 73.99 ± 0.82%, and 77.94 ± 0.90%, respectively. For mAP50–95, GSPN and ODA achieved the highest value of 33.62%, while RCMA reached 33.55 ± 0.69%, differing by only 0.07 percentage points from the best result.

Compared with GSPN, RCMA improved Recall and mAP50 by 3.22 and 1.73 percentage points, respectively. Relative to ODA, the corresponding gains were 2.54 and 0.97 percentage points, while RCMA also achieved higher values than FDAM across all five reported metrics. Overall, RCMA showed a stronger advantage in Recall and mAP50, whereas its advantage was less pronounced under the stricter IoU range represented by mAP50–95.

#### 2.1.3. Effectiveness Experiment of PGDAL

To evaluate different loss optimization strategies, MALoss, Varifocal Loss, and PGDAL were compared [28,29], as shown in Table 3. PGDAL achieved the highest mean values across all five reported metrics, with a precision of 74.94 ± 0.98%, a recall of 69.10 ± 1.64%, an F1 score of 71.90 ± 1.31%, an mAP50 of 76.33 ± 1.29%, and an mAP50–95 of 33.94 ± 0.49%. Compared with MALoss, PGDAL improved precision, recall, F1 score, mAP50, and mAP50–95 by 0.66, 0.65, 0.67, 0.62, and 1.24 percentage points, respectively. The differences were larger relative to Varifocal Loss, particularly for mAP50 and mAP50–95, which increased by 5.03 and 3.24 percentage points. Overall, the largest separation among the evaluated loss strategies was observed for mAP50–95.

### 2.2. Ablation Experiments

#### 2.2.1. Module Ablation Experiments

To separate the contribution of the shared image–text pathway from those of the three task-specific components, a TextGate-only configuration was included in the five-seed ablation experiment. As shown in Table 4, the visual-only Basic model achieved 68.33 ± 0.91% Recall, 70.23 ± 0.41% F1 score, 74.66 ± 0.60% mAP50, and 32.10 ± 0.39% mAP50–95. After introducing TextGate, Recall and F1 increased to 71.32 ± 1.61% and 72.01 ± 0.65%, respectively, while mAP50 increased slightly to 75.18 ± 0.48%. In contrast, mAP50–95 decreased to 31.32 ± 0.79%. Thus, the TextGate-only configuration showed higher mean Recall, F1, and mAP50 than Basic, but did not improve performance under the stricter IoU range.

Using TextGate as the common multimodal reference, adding TACIM, RCMA, and PGDAL increased mean mAP50 from 75.18% to 76.73%, 77.94%, and 76.33%, corresponding to numerical gains of 1.55, 2.76, and 1.15 percentage points, respectively. Their corresponding mAP50–95 values were 34.15%, 33.55%, and 33.94%, representing increases of 2.83, 2.23, and 2.62 percentage points relative to TextGate. These comparisons characterize the additional numerical contribution of each task-specific component under the same shared TextGate pathway.

Among the combined configurations, TextGate + TACIM + RCMA + PGDAL achieved the highest mean mAP50 of 79.52 ± 0.83% and an mAP50–95 of 34.26 ± 0.25%. Compared with TextGate + TACIM + RCMA, the strongest two-module configuration, adding PGDAL increased mAP50 from 78.93 ± 0.57% to 79.52 ± 0.83%, whereas mAP50–95 increased only from 34.14 ± 0.19% to 34.26 ± 0.25%. The corresponding additional gains were 0.59 and 0.12 percentage points. The smaller incremental gain in mAP50–95 indicates that the improvements of the three task-specific components were not strictly additive under more stringent localization criteria.

Table 5 lists the corresponding two-sided 95% confidence intervals for mAP50 and mAP50–95 obtained across the five random-seed training runs.

As shown in Table 6, the 3-epoch warmup achieved the highest mean performance across all five metrics. Compared with the 1-epoch setting, extending PGDAL supervision to three epochs increased mAP50 from 77.75% to 79.52% and mAP50–95 from 33.49% to 34.26%. Further extending the warmup to five epochs did not yield additional improvement, with mAP50 and mAP50–95 decreasing to 77.96% and 33.63%, respectively. These results support the use of PGDAL as a short early-training auxiliary constraint and indicate that three epochs provide an appropriate duration for the present configuration.

#### 2.2.2. Text Ablation Experiment

While Table 7 evaluates the presence or absence of the TextGate pathway within the network architecture, the following experiment keeps the complete TRP-Net architecture fixed and varies the textual input to examine the model response to different text contents.

The original-text condition achieved the highest mAP50 of 80.36%, exceeding the zero-text condition by 2.12 percentage points. Although the zero-text condition achieved a higher Precision of 78.30% than the 76.93% obtained with the original text, its Recall decreased from 74.12% to 71.23% and its F1 score decreased from 75.50% to 74.60%. The increase in Precision was accompanied by a decrease in Recall from 74.12% to 71.23%, indicating that the zero-text condition produced a more conservative detection behavior at the selected operating point. Without the matched textual semantics, predictions supported by weaker visual evidence were more likely to be suppressed, which can reduce false-positive responses and increase Precision, while also causing more true lesions to be missed. Consequently, the gain in Precision was achieved at the expense of detection coverage. Because mAP50 summarizes the precision–recall trade-off over the complete confidence range rather than at a single operating point, this shift resulted in a lower mAP50 of 78.24% compared with 80.36% for the original-text condition.

At the class level, the original-text condition increased EB AP50 from 72.01% to 73.18%, corresponding to an improvement of 1.17 percentage points, and increased LB AP50 from 84.46% to 87.54%, corresponding to an improvement of 3.08 percentage points. Neutral and shuffled text achieved mAP50 values of 79.43% and 79.76%, respectively, whereas removing coarse location information resulted in an mAP50 of 80.14% and an mAP50–95 of 34.59%. The original-text condition therefore yielded the highest mAP50 among the evaluated text settings, while the differences among the original, neutral, shuffled, and no-location conditions were relatively small. Because all text-input conditions were evaluated using a single fixed random seed, these differences are interpreted descriptively rather than as estimates of seed-independent effect size or statistical significance.

### 2.3. Detailed Performance Analysis

#### 2.3.1. Class-Specific Detection Performance

EB and LB achieved AP50 values of 73.18% and 87.54%, respectively, with corresponding AP50–95 values of 28.45% and 40.26%. The mean AP50 and AP50–95 across the two disease classes were 80.36% and 34.36%, respectively. The higher AP values for LB indicate that the model detected visible LB lesions more reliably than EB lesions. The larger decreases from AP50 to AP50–95 for both classes further indicate that precise bounding-box localization remained more challenging under stricter IoU thresholds. Table 8 presents these class-specific detection metrics.

#### 2.3.2. Scale-Specific Detection Performance

Table 9 presents the scale-specific detection performance of the original-text model on the validation set, with AP evaluated separately within each predefined lesion-size range. Small lesions accounted for 2513 of the 3358 annotated lesion instances, representing 74.84% of the evaluation data, whereas medium and large lesions accounted for 535 (15.93%) and 310 (9.23%) instances, respectively. The AP50 values for small, medium, and large lesions were 60.78%, 64.28%, and 61.23%, respectively, showing no monotonic increase with lesion size. Under the stricter IoU thresholds, AP50–95 increased from 19.40% for small lesions to 22.68% for medium lesions and 28.88% for large lesions. Thus, although AP50 showed a non-monotonic pattern across the three scale groups, AP50–95 was lowest for small lesions and increased with lesion size, indicating greater difficulty in precise localization of small lesions under stricter IoU criteria. Because small lesions occupy fewer pixels and retain less spatial detail after successive feature downsampling, even modest localization deviations can result in a relatively large reduction in bounding-box overlap, making their AP50–95 particularly sensitive to stricter IoU thresholds [30,31].

The overall mAP50 of 80.36% represents the arithmetic mean of the class-specific AP50 values for EB and LB evaluated over lesions of all sizes, whereas each scale-specific AP50 is recomputed within a restricted lesion-size subset. Because AP is derived independently from the precision–recall curve of each evaluation subset, the small-, medium-, and large-lesion AP50 values are not additive components of the overall mAP50 and are not expected to average to 80.36%.

#### 2.3.3. Precision–Recall Curves and Normalized Confusion Matrix

**Figure 3:**
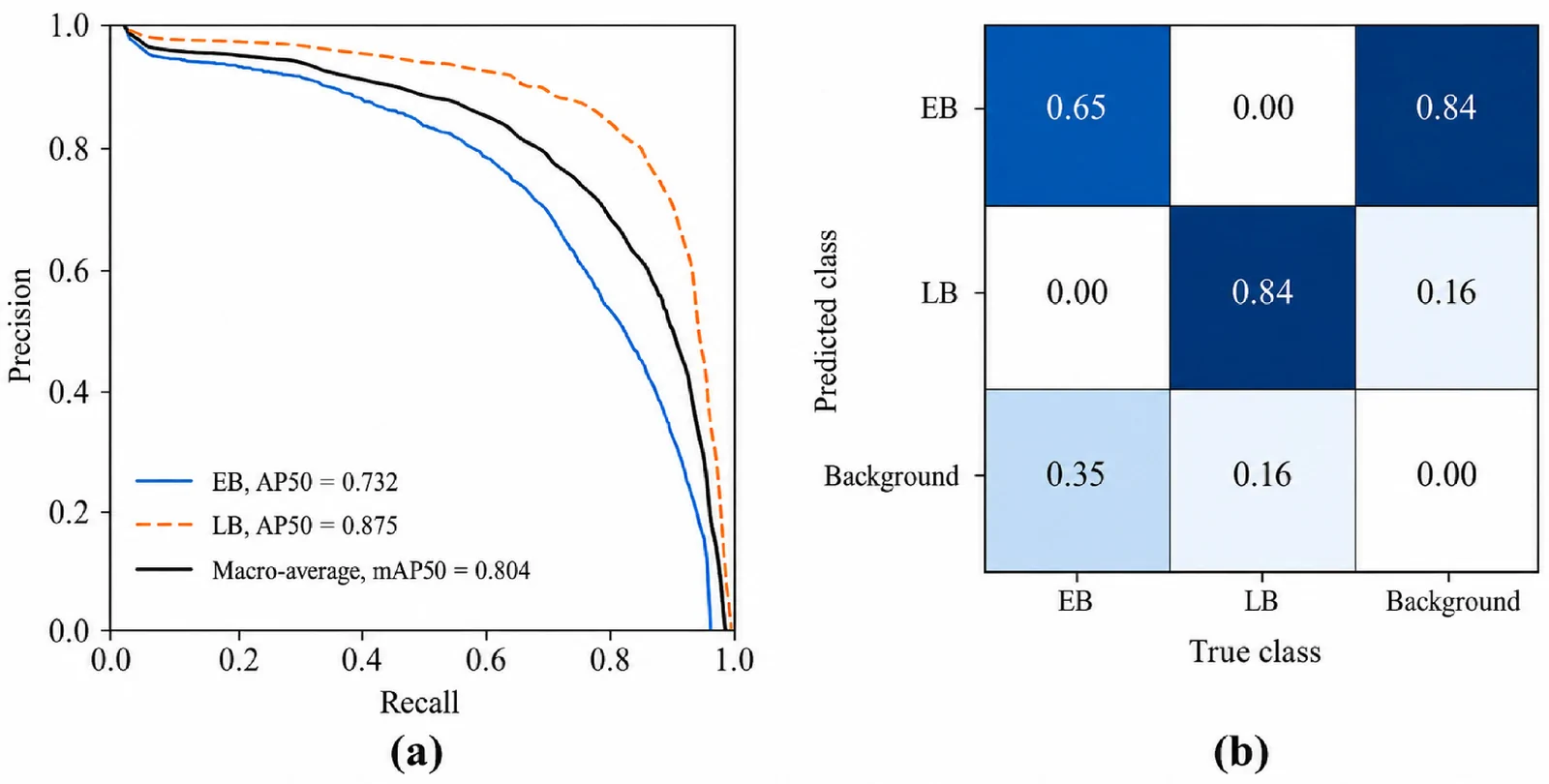
Class-wise detection performance of the original-text model on the validation set: (**a**) precision–recall curves for early blight and late blight; (**b**) normalized confusion matrix.

As shown in Figure 3a, the PR curve for LB remained above that for EB across most recall levels. LB achieved an AP50 of 87.5%, compared with 73.2% for EB, and the mean mAP50 across the two classes was 80.4%. The EB curve declined more sharply in the high-recall region, indicating that detecting additional EB lesions introduced more false positives and reduced precision.

The normalized confusion matrix is shown in Figure 3b. The correct detection proportions for EB and LB were 0.65 and 0.84, respectively, with almost no direct confusion between the two disease classes. Most errors involved confusion between lesions and the background. Specifically, 35% of EB lesions and 16% of LB lesions were not matched with valid predictions. Among predictions that did not match any ground-truth box, EB predictions accounted for a larger proportion than LB predictions.

### 2.4. Comparison Experiments on the Test Set

**Figure 4:**
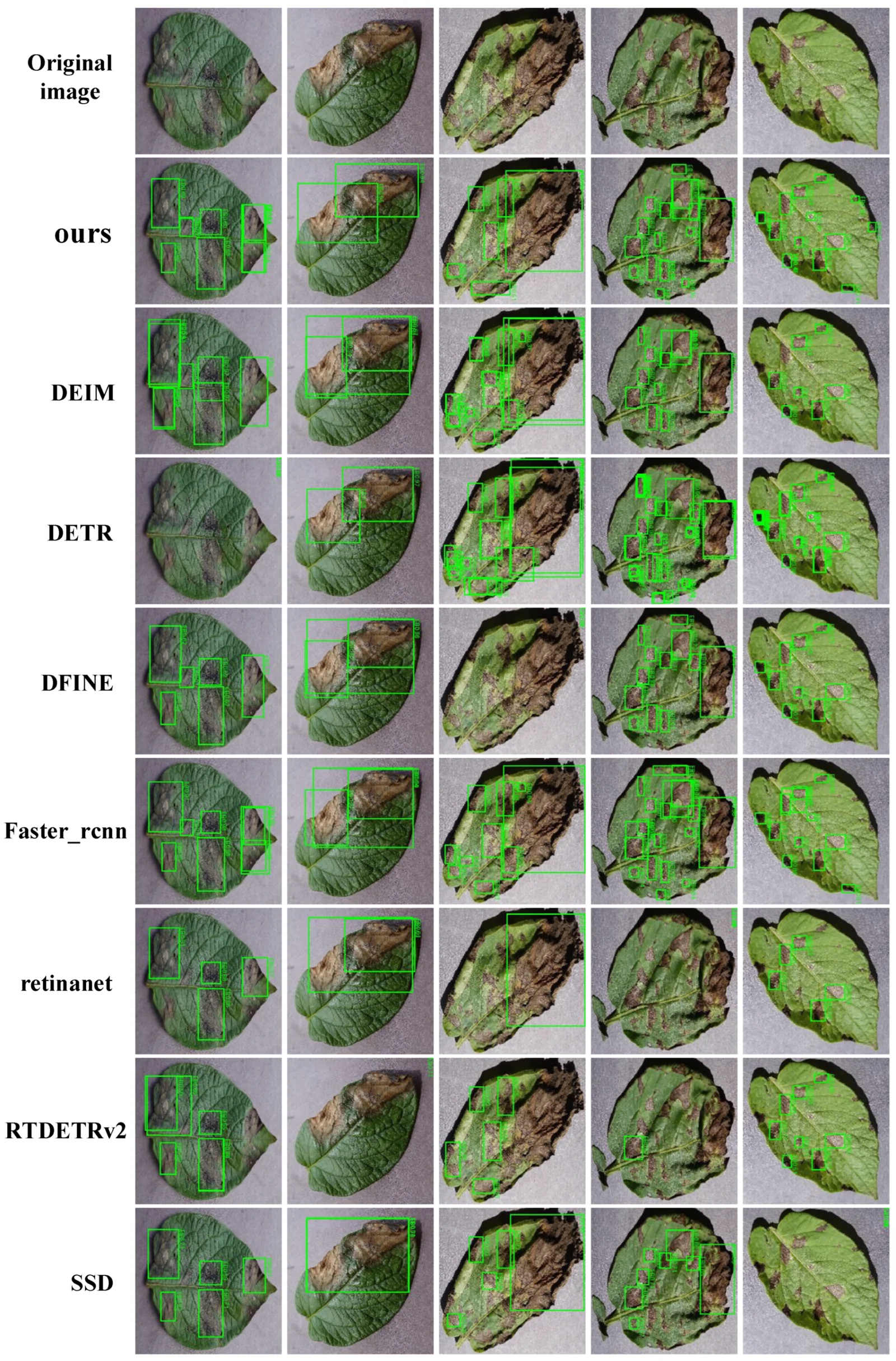
Qualitative comparison on five representative images from the independent potato test set. Columns 1–5 correspond to five fixed test images and are kept identical across all model rows. The first row shows the original RGB images; subsequent rows show predictions from TRP-Net, DEIM, DETR, D-FINE, Faster R-CNN, RetinaNet, RT-DETRv2, and SSD, respectively. All predicted bounding boxes are displayed using the same highlighted green color for visual clarity. Disease category is indicated by the EB or LB class label associated with each box; bounding-box color does not encode disease category. Only predictions with confidence scores ≥ 0.25 are visualized.

To evaluate the final detection performance of TRP-Net on the independent test set, the proposed method, TRP-Net, was compared with eight representative object detection models, namely YOLO11N, RT-DETRv2, D-FINE, Faster R-CNN, DEIM, RetinaNet, SSD, and DETR [32,33,34,35,36,37,38,39]. All models were trained and evaluated under the same data partitioning, input size, and training environment, with the experimental results shown in Table 10 and the visualized detection results shown in Figure 4. The quantitative results show that TRP-Net achieved 78.3% Precision, 73.7% Recall, 75.9% F1 Score, and 80.5% mAP50, obtaining the best overall performance. The independent-test mAP50 of 80.5% was slightly higher than the five-seed validation mean of 79.52 ± 0.83% reported in Table 4. The two values represent different evaluation summaries: Table 4 reports the mean across five independently trained checkpoints on the validation set, whereas Table 10 reports the final selected model on the independent test set. The validation set contains 2882 EB and 476 LB lesion instances, whereas the test set contains 1213 EB and 849 LB instances. Differences in source domain, class composition, and within-class sample difficulty may therefore contribute to the small numerical difference between the two evaluations. Because mAP50 is calculated as the arithmetic mean of class-wise AP50 values, the different EB/LB frequencies should not be interpreted as a direct instance-weighting effect.

As a direct comparison within the YOLO family, YOLO11N achieved a Precision of 72.8%, Recall of 70.3%, mAP50 of 73.3%, and mAP50–95 of 30.3%. TRP-Net exceeded YOLO11N by 7.2 percentage points in mAP50 and 4.3 percentage points in mAP50–95. This result indicates that the proposed modifications provide additional improvements in lesion detection and localization over the recent YOLO baseline under the present experimental setting. Specifically, compared with RT-DETRv2, which achieved the best mAP50 among the competing models, TRP-Net improved mAP50 by 6.7 percentage points; compared with D-FINE, which obtained the highest F1 Score among the comparison models, TRP-Net improved F1 Score by 3.7 percentage points, indicating that the proposed method achieves a better balance between lesion recognition accuracy and recall capability. At the detector side, TRP-Net contains 2.79 M parameters and requires 5.2 GFLOPs, maintaining relatively low model complexity while achieving the highest overall detection performance among the evaluated models. However, TRP-Net additionally uses reviewed image-level textual features, whereas the comparison models use only image inputs. Therefore, the reported Params and GFLOPs reflect detector-side architectural complexity rather than the end-to-end computational cost of the complete multimodal pipeline.

As shown in Figure 4, the compared models exhibited different detection responses across lesions with varying scales and background textures. In the first example, the lesions were small and scattered, with several regions showing visual similarity to surrounding leaf veins. DETR produced few valid detections, while several other models missed parts of the scattered lesions or generated relatively coarse bounding boxes. TRP-Net detected a larger proportion of the visible small lesions distributed across the leaf.

In the third example, a large necrotic region coexisted with several smaller grayish-brown lesions. Some comparison models mainly detected the visually prominent large lesion while missing smaller surrounding regions. In addition, several bounding boxes produced by Faster R-CNN and SSD extended beyond the visually apparent lesion boundaries. TRP-Net detected both the large necrotic region and multiple smaller lesions at different locations.

To further evaluate the performance of TRP-Net under natural-scene conditions, the natural-scene images in the independent test set were evaluated separately. As shown in Table 11, TRP-Net achieved an mAP50 of 79.1% and an mAP50–95 of 33.7% on the natural-scene subset. These results show that TRP-Net maintained relatively stable detection performance under the evaluated natural-scene conditions.

### 2.5. Architecture Transferability Experiment

To evaluate the transferability of the proposed architecture to another crop disease detection task, additional experiments were conducted on a tomato leaf disease dataset derived from PlantVillage. As summarized in Table 12, the dataset contained 2652 images of early blight (EB) and late blight (LB), with a total of 7096 annotated lesion instances. The predefined training, validation, and test subsets contained 1551, 539, and 562 images, respectively. The corresponding lesion counts were 3375, 1367, and 2354, comprising a total of 4532 EB and 2564 LB lesions. The lesion annotation procedure and the LLaVA-based image-level text generation and review protocol followed the same procedures as those used for the potato dataset.

For the tomato dataset, image-level descriptions were generated independently from the corresponding tomato RGB images using the same fixed prompt and manual-review criteria described in Section 2.1. The prompt requested only directly observable lesion attributes, including number, color, shape, and approximate position, without specifying the crop species or disease diagnosis. Therefore, potato descriptions or cached potato text features were not reused in the tomato experiment; instead, new tomato-specific descriptions and corresponding BERT features were generated before model training. TRP-Net and all comparison models were retrained on the tomato training set, validated on the corresponding validation set, and evaluated on the held-out test set using the same training and evaluation settings as in the potato experiments. Therefore, this experiment evaluates architecture transferability rather than zero-shot cross-dataset generalization.

Although the backgrounds of the PlantVillage tomato images are relatively standardized, the lesions still exhibit substantial variations in scale, low color contrast, irregular boundary shapes, and interference from leaf vein textures, providing an additional test of the applicability of the proposed architecture to a different crop disease dataset. TRP-Net was compared with representative object detection models, including YOLO11n, DEIM, DETR, D-FINE, Faster R-CNN, RetinaNet, RT-DETRv2, and SSD, as shown in Table 13.

**Table 13:** Architecture transferability results on the tomato leaf disease dataset.

Models	Precision	Recall	F1	mAP50	mAP50–95	Params (M)	GFLOPs
Ours	74.5	71.2	72.8	77.7	34.3	2.79	5.2
YOLO11n	66.6	61.4	63.9	66.1	27.2	2.6	6.5
DEIM	55.2	46.1	50.2	46.8	18.2	3.7	7.1
DETR	46.4	46.7	46.6	35.6	11.7	41.5	86.0
D-FINE	72.6	52.6	61.0	61.4	27.4	4.7	7.1
Faster R-CNN	69.3	56.3	62.1	65.2	26.9	41.3	133.9
RetinaNet	58.8	56.3	57.5	58.1	24.4	32.2	126.9
RT-DETRv2	69.5	58.8	63.7	65.1	28.3	36.0	120.0
SSD	67.7	55.4	60.9	60.6	26.1	23.8	30.4

Note: P, Precision; R, Recall; F1, F1 score; mAP50, mean average precision at an intersection-over-union threshold of 0.50; mAP50–95, mean average precision averaged over intersection-over-union thresholds from 0.50 to 0.95; Params, number of model parameters; M, million; GFLOPs, giga floating-point operations. P, R, F1, mAP50, and mAP50–95 are reported as percentages. Qualitative detection examples are shown in Figure 5, which further demonstrates that the TRP-Net architecture can be retrained for the evaluated tomato lesion-detection task.

**Figure 5:**
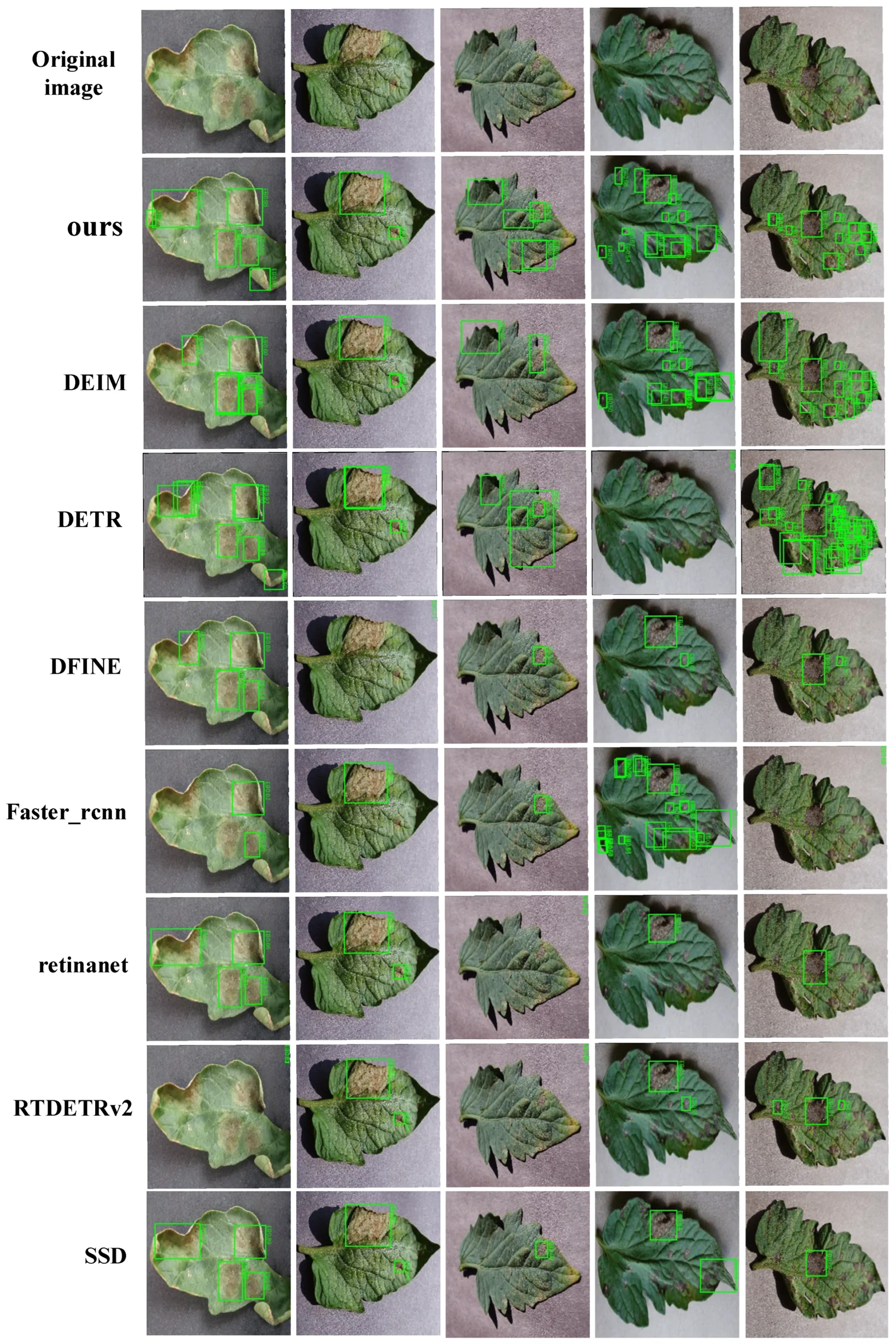
Qualitative comparison on five representative images from the held-out tomato test set. Columns 1–5 correspond to the five selected test images. Rows show the original RGB images followed by predictions from TRP-Net, DEIM, DETR, D-FINE, Faster R-CNN, RetinaNet, RT-DETRv2, and SSD, respectively. All predicted bounding boxes are displayed in green for visual clarity. Disease categories are indicated by the EB or LB class labels associated with the corresponding boxes; bounding-box color does not encode disease category. Only predictions with confidence scores ≥ 0.25 are visualized).

In this single-run transferability experiment, TRP-Net achieved 74.5% Precision, 71.2% Recall, 72.8% F1 score, 77.7% mAP50, and 34.3% mAP50–95. Among the comparison models, YOLO11n achieved the highest mAP50 of 66.1% and F1 score of 63.9%. TRP-Net showed numerical differences of 11.6 and 8.9 percentage points in these metrics, respectively. These results provide descriptive evidence that the TRP-Net architecture can be retrained for the evaluated tomato lesion-detection task.

## 3. Discussion

The results indicate that TACIM, RCMA, and PGDAL address different but partly overlapping aspects of potato lesion detection. TACIM mainly contributes to representation stability under environmental appearance variation, RCMA strengthens lesion-related feature discrimination and recall, and PGDAL provides additional position-aware supervision. The larger improvement observed in mAP50 than in mAP50–95 suggests that the combined design contributes more strongly to lesion recognition and coarse localization than to precise bounding-box fitting under stricter IoU criteria.

Environmental appearance variation remains an important source of performance degradation in lesion detection [38,39,40]. Recent studies have addressed low-light disease detection through illumination-aware feature learning and spatial reconstruction, with low-light representations helping preserve lesion information under reduced visibility [41]. Recent studies on field plant disease recognition have also shown that variations in illumination and acquisition conditions can produce domain shifts that affect disease-recognition performance [38,40]. In the present low-light evaluation, the mAP50 gap between Basic and Basic + TACIM increased from 0.78 percentage points under the original brightness condition to 5.13 percentage points under the strongest brightness reduction. This trend is consistent with the intended role of TACIM in constraining representation changes across environmentally perturbed views. However, the current experiment considers only controlled brightness reduction and does not cover the full range of field variations, such as combined illumination shifts, occlusion, blur, or device-dependent appearance changes. Therefore, the results support improved robustness to the evaluated illumination perturbation, while broader field robustness requires further validation.

The TextGate-only and text-input experiments together provide a more nuanced view of the role of textual semantics in lesion detection. The TextGate-only configuration modestly increased mAP50 and produced clearer gains in Recall and F1 relative to the visual-only Basic model, while its mAP50–95 was lower. In the present single-seed analysis, correctly matched descriptions achieved an mAP50 that was 2.12 percentage points higher than that of the zero-text condition. However, the neutral and shuffled descriptions remained relatively close to the original-text condition, while removing coarse location information did not reduce mAP50–95. These observations suggest that the contribution of textual information may depend on its semantic relevance and interaction with visual features rather than merely on the presence of an additional modality [21,22,23,24]. Dong et al. [40] similarly reported limitations of text-guided multimodal models in fine-grained plant disease anomaly detection and emphasized the importance of visual information for distinguishing subtle disease patterns. Wang et al. [41] further highlighted the challenge of aligning general multimodal representations with crop disease characteristics and introduced domain adaptation and cross-modal alignment to improve disease-specific representation. More broadly, Shafay et al. [42] identified effective multimodal fusion and cross-modal consistency as continuing challenges in plant disease analysis. In this context, the present results indicate that textual descriptions in TRP-Net can provide complementary semantic cues for lesion discrimination, whereas precise spatial localization remains more strongly dependent on visual and position-sensitive representations [42,43,44].

Small lesions generally contain limited spatial detail and are particularly susceptible to information loss during successive downsampling [30,31]. Precise lesion localization remains an important limitation of TRP-Net. Although the independent-test mAP50 reached 80.5%, the corresponding mAP50–95 was 34.6%, indicating a substantial performance reduction as the overlap criterion became stricter. This limitation was particularly evident for small lesions: small-lesion AP50–95 was 19.40%, compared with 22.68% for medium lesions and 28.88% for large lesions. Recent plant disease detectors have addressed similar scale-related difficulties through explicit multiscale feature propagation and dedicated small-target detection heads [45,46]. TomaFDNet emphasizes multiscale feature diffusion for small lesions in complex backgrounds, whereas TomatoLeafDet introduces high-resolution multiscale fusion and a specialized small-target detection head. Although cross-study performance values are not directly comparable because of differences in datasets and evaluation protocols, these designs highlight the importance of preserving high-resolution spatial information for precise lesion localization. In TRP-Net, PGDAL provides position-distribution supervision during early training, but it does not explicitly model lesion contours or introduce an additional high-resolution detection head. This may partly explain why localization gains remain limited under stricter IoU criteria.

The ablation results also show that the effects of the three modules are not simply additive. TACIM + RCMA achieved an mAP50–95 of 34.14 ± 0.19%, whereas adding PGDAL increased this value only to 34.26 ± 0.25%. A similar pattern was observed across several two-module combinations, suggesting diminishing incremental gains and possible functional overlap among the modules. TACIM, RCMA, and PGDAL operate at different stages, but all ultimately influence the quality of lesion representations used by the same detection head. Improvements in feature stability, semantic discrimination, and spatial response may therefore converge on overlapping error cases. The complete model nevertheless achieved the highest overall mean performance, suggesting complementary effects at the system level, while the small incremental gain in mAP50–95 indicates diminishing returns for strict localization.

A clear class-dependent performance gap was also observed between early blight and late blight. EB achieved an AP50 of 73.18% and an AP50–95 of 28.45%, compared with 87.54% and 40.26% for LB, respectively. The normalized confusion matrix showed a consistent pattern, with 35% of EB lesions remaining unmatched compared with 16% of LB lesions. A similar class asymmetry has been reported in recent tomato disease detection research, where TomaFDNet obtained lower AP for early blight than for late blight [44], although direct comparison is limited by differences in crop species, datasets, and evaluation settings. In the present dataset, the weaker EB performance cannot be explained solely by a shortage of EB annotations, suggesting that lesion appearance and localization difficulty are also important factors. The sharper decline of the EB precision–recall curve at high recall indicates that recovering additional EB lesions is accompanied by more false responses. Visually similar leaf textures, weak lesion contrast, and small or scattered lesion patterns may contribute to this behavior [47,48,49], although their individual effects cannot be separated in the current experiments. The persistence of the EB–LB gap despite position-aware auxiliary supervision indicates that EB-specific detection errors remain unresolved.

TRP-Net is mainly intended for the screening of leaves with visible abnormal symptoms and for marking suspected early blight and late blight lesion regions. Its outputs may help identify areas that require closer inspection or sampling. The detector-side inference network contains 2.79 M parameters and requires 5.2 GFLOPs, indicating low computational complexity for lesion detection. Recent lightweight plant disease detection studies have evaluated practical deployment directly on embedded platforms using device-level inference metrics rather than model complexity alone [50]. These values exclude LLaVA-based text generation, BERT text encoding, and the offline manual review process. End-to-end latency and memory usage of the complete image–text pipeline were not evaluated. Therefore, the results support the lightweight design of the detector-side network but do not establish lightweight or real-time performance of the complete multimodal pipeline. At its current stage, TRP-Net should be regarded as an auxiliary method for visible-symptom screening, suspected-lesion localization, and expert review rather than as an independently deployed real-time diagnostic system.

This study has several limitations. First, the dataset was assembled from multiple public sources, and some images lacked standardized information on cultivar, growth stage, geographic location, acquisition device, and pathogen confirmation. Previous field-based studies have shown that variations in illumination, acquisition conditions, crop characteristics, and deployment environments can produce domain shifts in plant disease recognition [39,46,50]. The current results therefore cannot provide a strict evaluation of generalization across regions, cultivars, and devices. Moreover, because the independent potato test set was constructed from different public sources than the training and validation sets, source-domain differences and class-composition differences are jointly reflected in the reported test performance and cannot be disentangled in the current dataset. Future work should establish field datasets with complete metadata across locations, cultivars, and years. Second, the present RGB-based framework is restricted to the recognition of visible symptom patterns and was not designed to identify latent infections before visible symptoms appear. Optical sensing technologies, particularly hyperspectral and other spectral sensing approaches, have been investigated for early plant disease diagnosis and may provide complementary information beyond conventional visible-symptom analysis [51]. Future studies could incorporate multispectral, thermal infrared, or other physiological sensing data. Finally, the model has not yet been validated on low-power edge devices. Recent lightweight plant disease detection studies have evaluated their models directly on embedded platforms to characterize practical inference efficiency and deployment feasibility [51,52]. Future work will evaluate end-to-end latency, peak memory use, power consumption, and deployment performance on low-power edge devices. These limitations are consistent with the challenges of dataset bias, environmental variation, cross-domain generalization, multimodal sensing, and edge deployment reported in previous plant disease studies [39,43,46,50,51,52,53].

In summary, this study developed a text-guided lesion-detection framework and examined environmental appearance stability, image–text semantic assistance, and position-aware supervision for small lesions. Evidence was obtained from five-seed controlled ablation experiments, low-light perturbation tests, text-input ablations, and an architecture-transferability experiment. Relative to the shared TextGate pathway, TACIM, RCMA, and PGDAL produced different numerical gains, and their combination achieved the highest mean mAP50. Further improvement remains necessary for precise localization under stricter IoU criteria.

## 4. Materials and Methods

### 4.1. Datasets

This study integrated four publicly available potato leaf image resources to construct the dataset. These sources included PlantVillage, the Potato Disease Leaf Dataset (PLD) released on Kaggle, and the Potato Computer Vision Model and Healthy Leaf Dataset from Roboflow Universe. Detailed source information is provided in Table 14.

After image collection, source verification, host-plant review, and quality screening. After screening, 3004 images were retained, and their source-level distribution across the training, validation, and test sets is summarized in Table 15.

After image collection, source verification, host-plant review, and quality screening were conducted using consistent criteria. Image clarity, resolution, leaf integrity, class reliability, and watermark obstruction were examined. Images were excluded when they were severely blurred, difficult to identify, uncertain in host species, or heavily obscured in key regions. Visible lesions associated with EB and LB were then manually annotated using LabelImg (version 1.8.6). Healthy images were manually confirmed to contain no visible EB- or LB-related lesions and were included as target-free negative samples with zero lesion bounding boxes. All screening decisions, class labels, and bounding-box annotations were independently reviewed by two examiners. Disagreements were resolved through joint reassessment, and samples with insufficient evidence of disease symptoms were excluded. After screening, 3004 images were retained, and their source-level distribution across the training, validation, and test sets is summarized in Table 15. Because some images came from public datasets, complete provenance metadata and original camera files were unavailable for all samples. Prior digital editing therefore could not be verified in every case.

Images were used as the basic sampling unit for dataset partitioning and model training. Because the public repositories did not consistently preserve unique identifiers for individual plants or leaves, the exact numbers of independent plants and leaves could not be reliably reconstructed. Dataset statistics were therefore reported at two levels to avoid conflating different sampling units. At the image level, the numbers of EB, LB, and Healthy images were recorded for each data split. At the annotation level, each bounding box was treated as one visible lesion instance, and EB and LB bounding boxes were counted separately. Healthy images contained no visible lesions associated with EB or LB and were included as target-free negative samples with zero bounding boxes. The complete dataset comprised 3004 images, including 2100 training images, 454 validation images, and 450 test images, representing 69.91%, 15.11%, and 14.98% of the dataset, respectively. The image-level class distribution and annotation-level lesion-instance distribution are summarized in Table 16.

Although the image-level numbers of EB and LB were relatively similar, the annotation-level lesion distribution was imbalanced. EB and LB accounted for approximately 79.2% and 20.8% of the annotated lesion instances, respectively, corresponding to an EB-to-LB ratio of approximately 3.80:1. Therefore, in addition to overall mAP, class-specific AP values were reported to provide a more transparent evaluation of category-level performance.

Based on directly observable scene characteristics, the retained images were further categorized into controlled-background and natural-scene groups. Controlled-background images generally contained a single or isolated leaf against a relatively uniform background, whereas natural-scene images included complex factors such as soil, stems, weeds, overlapping leaves, partial occlusion, and uneven illumination. The distribution of these scene categories across the training, validation, and test sets is summarized in Table 17.

The training and validation sets were randomly split at the image level from a development pool containing PlantVillage and PLD, whereas the independent test set was constructed from unused Roboflow Universe projects, as reflected in Table 15. This source-level separation introduces a potential domain shift between the development and test sets because the images originate from different public repositories and may differ in acquisition conditions, background characteristics, and sample composition. In addition, the validation and test sets differed in disease-class composition (Table 16) and scene composition (Table 17). Therefore, the independent test set was used to assess performance under a source-shifted evaluation setting rather than as an identically distributed extension of the development data. To prevent data leakage across subsets, exact and near-duplicate checking was performed for all three partition pairs (Train–Val, Train–Test, and Val–Test) using SHA-256 hashing, pixel-level comparison after EXIF correction and RGB decoding, and 64-bit perceptual hashing. Image pairs with a pHash Hamming distance of 5 or less were further reviewed by two examiners, and no confirmed cross-partition duplicates were identified.

Original images were retained, and no fixed-area cropping was applied. Each complete image was resized to fit within 768 × 768 pixels using letterbox preprocessing with bilinear interpolation. The original aspect ratio was preserved, and padding with RGB values of (114, 114, 114) was added when required. Bounding-box coordinates were transformed using the same scaling factor and padding offsets. This procedure standardized the detector input resolution while preserving the original geometric relationships of the annotated lesion regions.

Following dataset partitioning and image preprocessing, the base LLaVA v1.6 model [49] was deployed via Ollama to generate one image-level description for each RGB image. A fixed prompt was used for all images: “Describe leaf lesions in one sentence. Note count, color, shape and position. Do NOT diagnose disease. Examples: A small brown, irregularly shaped lesion is visible in the lower-right region of the leaf.” The prompt restricted the generated descriptions to directly observable lesion characteristics, including lesion number, color, shape, and approximate position, rather than disease diagnosis. Disease labels, bounding-box annotations, and other annotation-derived information were not provided to LLaVA during text generation.

All LLaVA-generated descriptions were independently reviewed by two examiners using only the corresponding RGB images and generated text, without access to EB/LB labels or bounding-box annotations. Both examiners followed the same predefined correction criteria throughout the review process, and corrections were limited to inconsistencies between the generated descriptions and visually observable lesion characteristics. The finalized descriptions retained only observable attributes, including lesion number, color, shape, and approximate spatial distribution, while disease names, class labels, exact coordinates, and annotation-derived information were excluded. The reviewed descriptions were finalized before model training and remained unchanged throughout all experiments. LLaVA-based description generation and manual review were conducted as offline preprocessing steps and were excluded from the detector-side inference evaluated in this study. Each image was paired with one reviewed description, and no default substitute text was used. Representative examples of the original and manually corrected descriptions are provided in Table 18.

**Figure 6:**
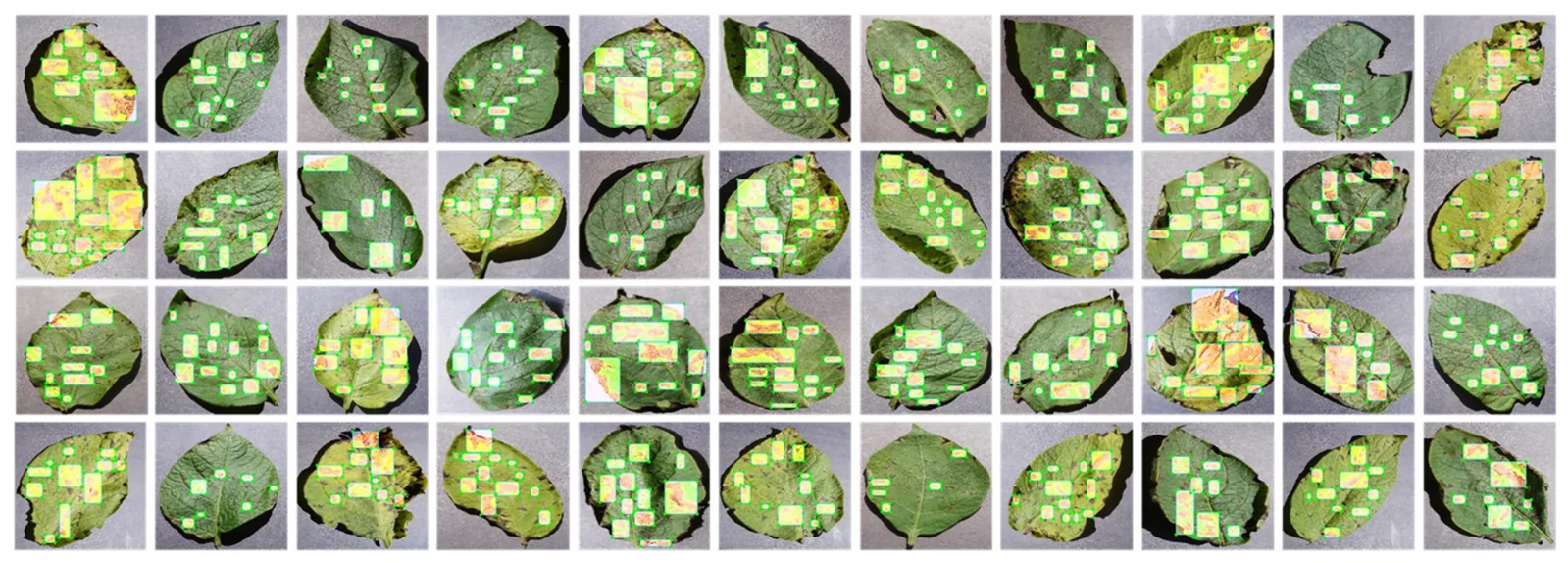
Representative lesion annotations from the training, validation, and test subsets of the potato dataset. Bounding boxes denote manually annotated early blight (EB) and late blight (LB) lesions. Different box colors only reflect visualization differences after annotation and do not indicate different lesion categories..

Consequently, although exact and near-duplicate images were excluded across data splits, residual dependence among different images originating from the same plant or leaf cannot be completely ruled out. Representative annotated samples from the potato dataset are presented in Figure 6. Note that although exact and near-duplicate images were excluded across data splits, residual dependence among different images originating from the same plant or leaf cannot be completely ruled out.

### 4.2. Baseline Network and Model Motivation

YOLO26n was selected as the baseline detector, and the original YOLO26n model is referred to as Basic [50]. The baseline implementation was based on Ultralytics v8.4.50 (official repository: https://github.com/ultralytics/ultralytics (accessed on 10 September 2026); release tag: v8.4.50), using the n-scale YOLO26 configuration and COCO-pretrained YOLO26n weights. YOLO26n was selected because its lightweight n-scale architecture provides P3/8, P4/16, and P5/32 multiscale features while maintaining relatively low detector-side complexity, making it suitable for lesions with substantial scale variation and providing a baseline for evaluating the additional modules proposed in this study. Three-channel RGB images were resized to 768 × 768 pixels before being fed into the network. The neck generated P3/8, P4/16, and P5/32 feature maps with 64, 128, and 256 channels, respectively, and the detection head was configured for two lesion classes, namely early blight and late blight. Basic retained the original YOLO26n backbone, neck, and detection head. It follows a one-stage detection framework comprising a backbone, a neck, and a detection head. Its low model complexity and high inference efficiency make it suitable for multiscale potato lesion detection. The backbone extracts hierarchical visual features through convolutional layers and C3k2 modules. Shallow features retain spatial details such as lesion color, texture, and edges, whereas deeper features encode disease-related semantic information. The neck performs bidirectional feature fusion through upsampling, feature concatenation, and downsampling, producing multiscale P3, P4, and P5 feature maps. Based on these fused features, the detection head predicts class confidence scores and bounding boxes for early blight and late blight lesions, enabling lesion classification and localization at different scales.

Potato leaf lesions vary considerably in scale, number, and spatial distribution, requiring both detailed spatial information and higher-level semantic representations. The P3, P4, and P5 feature maps of YOLO26n provide complementary multiscale features for representing small scattered lesions, connected lesions, and larger necrotic regions, while maintaining relatively low model complexity. However, the baseline detector relies primarily on visual information and may be affected by illumination variation, shadows, blur, lesion-like leaf textures, and the loss of spatial details during successive downsampling. To address these limitations, TRP-Net first employs TextGate as a shared image–text fusion interface to incorporate image-level semantic information into visual features. Based on this common multimodal pathway, three task-specific components, TACIM, RCMA, and PGDAL, are introduced to improve feature stability under environmental variation, suppress interference from lesion-like background patterns, and enhance small-lesion localization, respectively. Accordingly, the ablation design treats TextGate as the shared multimodal reference. Basic is used as the visual-only baseline, TextGate isolates the effect of introducing the image–text pathway, and TACIM, RCMA, and PGDAL are evaluated individually and in all factorial combinations on top of the same TextGate pathway.

### 4.3. TRP-Net Network Structure

**Figure 7:**
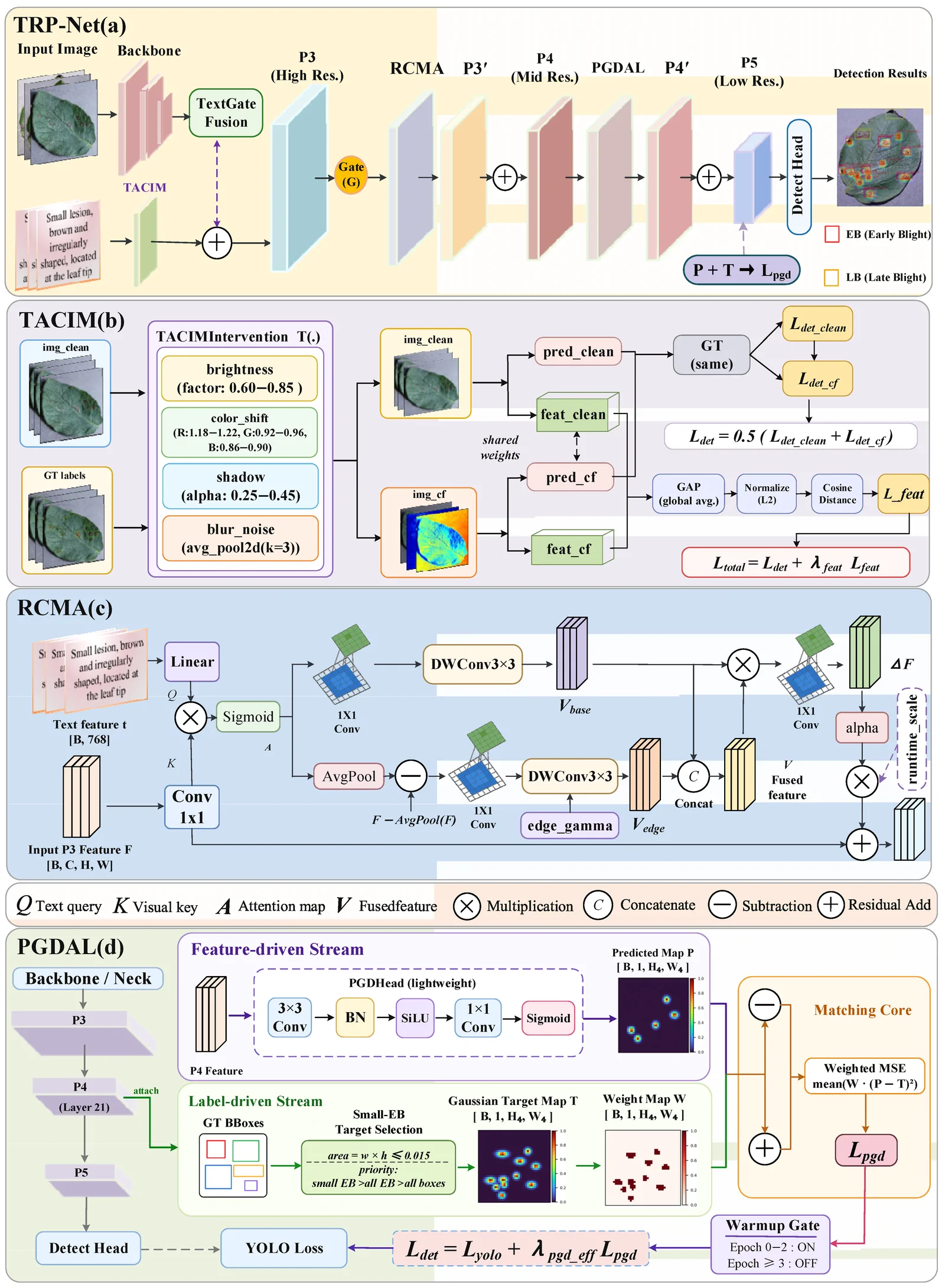
Architecture of TRP-Net and its main components: (**a**) overall network; (**b**) TACIM; (**c**) RCMA; and (**d**) PGDAL. RCMA, PGDAL, and TextGate operate at the P3/8, P4/16, and P5/32 feature stages, respectively. TACIM and PGDAL are training-time components. Colors are used only for visualization purposes and do not indicate different lesion categories.

Built upon YOLO26, TRP-Net incorporates three dedicated components, namely TACIM, RCMA, and PGDAL, to improve potato leaf disease detection under complex field conditions while maintaining low detector-side model complexity, as illustrated in Figure 7. The overall pipeline is as follows. First, the input image and its corresponding textual description are encoded to obtain visual and semantic features. During training, TACIM generates environmentally perturbed counterfactual samples and enforces feature consistency between the original and perturbed images, thereby reducing the influence of illumination variation, shadow interference, color shift, and image blur. Subsequently, RCMA performs text-guided visual feature enhancement through cross-modal interaction, enabling the network to focus on lesion-relevant regions while strengthening boundary and fine-grained texture representations. Furthermore, PGDAL is introduced at the P4 feature level during the early training stage to provide Gaussian position distribution supervision, thereby improving the localization accuracy of small and weak lesions. Finally, the enhanced features are fused and forwarded to the detection head for disease classification and lesion localization.

For the textual branch, each reviewed image-level description was encoded using a locally archived pretrained BERT model. The encoder contains 12 Transformer layers and 12 attention heads, with a hidden dimension of 768 and a vocabulary size of 30,522. Text was processed using BertTokenizer with WordPiece tokenization and lower-case normalization. Sequences were padded or truncated to a maximum length of 128 tokens. The final hidden state of the [CLS] token, last_hidden_state [:, 0, :], was used as the 768-dimensional text feature. The BERT parameters were frozen during detector training. Text features were precomputed and cached to reduce repeated computation. The original pretrained-model identifier was unavailable; therefore, the architecture and tokenizer settings are reported explicitly.

The resulting 768-dimensional text feature is fused with the visual representation through TextGate. In the n-scale configuration used in this study, TextGate is inserted after the P5/32 feature stage. Let *X* ∈ ℝ*^B^*^×256×*H*/32×*W*/32^ denote the visual feature and *T* ∈ ℝ*^B^*^×768^ denote the corresponding image-level text feature. Two independent linear projections map the text feature from 768 dimensions to the 256 visual channels to generate text-conditioned scale and bias vectors:

*S* = tanh(*W_s_T* + *b_s_*) (1)

*B_t_* = tanh(*W_b_T* + *b_b_*) (2)

The strength of text modulation is controlled by a learnable bounded coefficient:

*α* = *α*_max_*σ*(*α*_logit_) (3)

and the TextGate output is calculated as:

*X^TG^* = *X*⊙(1 + *αS*) + *αB_t_* (4)

Here, *σ*(⋅) denotes the sigmoid function and ⊙ denotes element-wise multiplication. The projected scale and bias vectors are broadcast along the spatial dimensions. Thus, TextGate performs FiLM-style weak residual modulation through multiplicative channel-wise scaling and additive bias while retaining the original visual feature. TextGate does not use feature concatenation, normalization, or dropout, and its output has the same channel number and spatial resolution as the input. For the n-scale model with 256 P5 channels, TextGate contains 393,729 trainable parameters.

During training, the image–text pathway is jointly optimized with TACIM, RCMA, and PGDAL. TACIM performs clean–counterfactual consistency learning, RCMA enhances P3 features through cross-modal interaction, PGDAL provides auxiliary position-aware supervision at P4 during early training, and TextGate integrates textual information into the P5 feature. The overall architecture is shown in Figure 7.

### 4.4. TACIM

In complex field environments, potato leaf images are easily affected by variations in illumination intensity, color shifts, local shadows, imaging blur, and noise, causing lesions of the same category to exhibit obvious appearance differences under different acquisition conditions. Such environmental perturbations can alter the color distribution, texture response, and boundary clarity of lesion regions, causing the model to learn environment-related features that are irrelevant to disease, thereby reducing cross-scenario detection stability [51,52]. To address this issue, this study proposes TACIM, which guides the model to learn disease representations that are more stable under environmental variations through counterfactual environmental intervention and feature consistency constraints during training.

Given an original image *I*,text feature ***T***, and detection annotations *Y* = {*cls*,*bbox*,*bath*_−_*idx*}, TACIM generates a counterfactual image through an environmental intervention function **A***_env_*(∙):

*I_cf_* = **A***_env_*(*I*) (5)

Here, **A***_env_*(∙) represents a set of non-geometric environmental perturbations, including brightness scaling, color deviation via channel gain adjustment, local shadow simulation based on soft masks, and mean-blur augmentation with random noise. These operations only change the environmental appearance of the image and do not alter the lesion category or spatial location. Therefore, the counterfactual image shares the same detection annotation and text features as the original image:

***Y****_cf_* = ***Y***, (6)

***T****_cf_* = ***T*** (7)

It should be noted that “text-anchored” in TACIM does not mean that the generation process of counterfactual images is directly controlled by textual content. The generation of counterfactual images only affects the environmental appearance of the image; text anchoring is mainly reflected in the fact that the original image branch and the counterfactual image branch share the same text feature, and a consistency constraint is established in the feature space after TextGate fusion. In this way, the text feature provides a stable semantic reference for paired clean–counterfactual training, enabling the model to maintain stable disease semantic representations and visual feature expressions when the environmental appearance changes.

During training, the original image I and the counterfactual image *I_cf_* are separately fed into the detection network *f_θ_*(∙) together with the same text feature ***T***, producing two sets of detection predictions and post-TextGate features:

(*P_clean_*,*F_clean_*) = *f_θ_*(*I*,*T*) (8)

(*P_cf_*,*F_cf_*) = *f_θ_*(*I_cf_*,*T*) (9)

Here, *P_clean_* and *P_cf_* denote the detection outputs of the original-image branch and the counterfactual-image branch, respectively, while *F_clean_* and *F_cf_* represent the intermediate features of the two branches after textual semantic fusion. Since the two branches share the same annotations, the model computes detection supervision for the original and counterfactual images separately:

(10) $$L_{\det}^{\mathrm{clean}}=L_{\det}\left(P_{\mathrm{clean}},Y\right)$$

(11) $$L_{\det}^{\mathrm{cf}}=L_{\det}\left(P_{\mathrm{cf}},Y\right)$$

Although relying only on paired detection supervision can improve the model’s adaptability to different appearance conditions, it is still difficult to ensure that the disease representations learned inside the network remain consistent before and after environmental perturbation. Therefore, TACIM further introduces a consistency constraint on the post-TextGate features.

Specifically, global average pooling is first applied to compress the spatial feature maps into image-level semantic representations. The resulting vectors are then *L*_2_-normalized, and cosine distance is used to measure the feature discrepancy between the clean and counterfactual branches. To provide a stable semantic reference, the post-TextGate feature of the clean-image branch is detached using a stop-gradient operation. Gradients from the feature-consistency loss are therefore propagated only through the counterfactual branch, encouraging its representation to approach that of the clean branch under environmental perturbations. Let $F_{\mathrm{clean},i}^{\mathrm{TG}}$ and $F_{\mathrm{cf},i}^{\mathrm{TG}}\in R^{C\times H\times W}$ denote the post-TextGate features of the clean and counterfactual branches for the *i*-th sample, respectively. The spatial feature maps are compressed by global average pooling and then *L*_2_-normalized:

(12) $$z_{\mathrm{clean},i}=\mathrm{Norm}\left[\mathrm{GAP}\left(\mathrm{sg}\left(F_{\mathrm{clean},i}^{\mathrm{TG}}\right)\right)\right]$$

(13) $$z_{\mathrm{cf},i}=\mathrm{Norm}\left[\mathrm{GAP}\left(F_{\mathrm{cf},i}^{\mathrm{TG}}\right)\right]$$

The feature-consistency loss is then defined as:

(14) $$L_{\mathrm{feat}}=\frac{1}{B}\sum_{i=1}^{B}\left(1-z_{\mathrm{clean},i}^{T}z_{\mathrm{cf},i}\right)$$

Here, *B* denotes the batch size, GAP(⋅) denotes global average pooling, Norm(⋅) denotes *L*_2_ normalization, and *sg*(⋅) denotes the stop-gradient operation. Because the normalized vectors have unit length, their inner product is equivalent to cosine similarity. The stop-gradient operation is applied only to the clean branch, allowing it to serve as a fixed semantic reference while the counterfactual branch is optimized by *L*_feat_. This loss does not directly constrain bounding-box predictions or classification outputs; instead, it encourages stable semantic representations before and after environmental perturbation.

Finally, the training objective of TACIM consists of the original-image detection loss, the counterfactual-image detection loss, and the feature consistency loss:

(15) $$L_{\mathrm{TACIM}}=\frac{1}{2}\left(L_{\det}^{\mathrm{clean}}+L_{\det}^{\mathrm{cf}}\right)+\lambda_{\mathrm{feat}}L_{\mathrm{feat}}$$

Here, *λ_feat_* is the weight of the feature consistency loss, which is set as follows in this study:

*λ_feat_* = 0.005 (16)

Consistency regularization has been widely used to encourage stable representations across perturbed views of the same input [53,54]. In TACIM, *L*_feat_ is defined by the cosine distance between *L*_2_-normalized feature vectors and is therefore bounded between 0 and 2. Accordingly, *λ*_feat_ = 0.005 was used to keep this consistency term as a weak auxiliary regularizer while preserving the primary role of the detection objective.

Through the above design, TACIM explicitly incorporates field environmental variations into the training process and establishes paired supervision between the original and counterfactual images. The detection loss ensures that the model can perform disease recognition and localization under both appearance conditions, while the feature consistency loss further constrains the network to maintain stable representations in the feature space after textual semantic fusion. Therefore, TACIM can guide the model to maintain more stable disease-related representations before and after environmental perturbations, reducing its dependence on non-disease factors such as illumination, shadows, color shifts, and blur noise.

After training, TACIM no longer generates counterfactual images or computes the feature consistency loss. During inference, the model follows the main image–text detection pathway to complete prediction. Thus, the role of TACIM is concentrated on counterfactual supervision and representation constraints during training, providing a more stable feature basis for potato leaf disease detection in complex field environments while maintaining a concise inference process.

### 4.5. RCMA

Early lesions on potato leaves are usually characterized by small scales, weak boundary responses, and subtle texture differences. In complex field environments, leaf veins, shadows, leaf folds, and natural textures may produce local responses similar to lesion regions, resulting in insufficient feature representation of small lesions and false activation of background areas. Although high-resolution shallow features retain rich spatial details, they are also more susceptible to interference from non-disease textures [55,56]. Therefore, this study introduces an RCMA into the P3/8 small-object detection branch to recalibrate P3 features before they are fed into the detection head, thereby enhancing the semantic discriminability and weak boundary responses of small-lesion-related regions.

Given the P3-branch visual feature *F* ∈ ℝ*^B^*^×*C*×*H*×*W*^ and the image-level textual feature *T* ∈ ℝ*^B^*^×768^ RCMA first uses the textual feature to generate a semantic query vector:

*Q* = *W_q_T*; *Q* ∈ ℝ*^B^*^×*d*×1×1^ (17)

Here, *W_q_* denotes the textual feature mapping matrix, and *d* represents the attention dimension. Meanwhile, the visual feature is mapped into the visual key feature through a 1 × 1 convolution:

***K*** = *Conv*_1×1_(***F***), ***K*** ∈ ℝ*^B^*^×^*^d^*^×^*^H^*^×^*^W^* (18)

Through element-wise interaction between the textual query *Q* and the visual key ***K***, the module forms attention weights modulated by textual semantics over spatial visual responses, ensuring that subsequent feature enhancement is constrained by image-level disease semantics rather than being determined only by local texture intensity.

During value-feature construction, RCMA models the basic visual branch and the high-frequency residual branch in parallel. The base visual branch is used to preserve the original local textures and contextual responses:

*V_base_* = DWConv_3×3_(*Conv*_1×1_(*F*)) (19)

Here, DWConv_3×3_ denotes a 3 × 3 depthwise convolution. Considering that early lesion edges, necrotic textures, and abrupt color changes usually appear as local high-frequency variations, RCMA further constructs boundary-sensitive features using local mean residuals:

*F_edge_* = *F* − *AvgPool*(*F*) (20)

This operation suppresses smooth low-frequency responses and highlights local texture changes and weak boundary information. Subsequently, the high-frequency residual features are processed by a lightweight convolutional branch to obtain boundary-aware value features:

*V_edge_* = *γ_e_* ⋅ DWConv_3×3_(*Conv*_1×1_(*F_edge_*)) (21)

Here, *γ_e_* is the intensity modulation coefficient of the high-frequency residual branch, which controls the contribution of boundary-sensitive information during feature fusion. It should be noted that Boundary in this study refers to boundary-aware feature enhancement based on high-frequency residuals and does not involve explicit boundary annotations or boundary loss.

The base visual branch and the high-frequency residual branch are then concatenated and passed through a 1 × 1 convolution to obtain the fused value feature:

*V* = *Conv*_1×1_([*V_base_*,*V_edge_*]) (22)

In the cross-modal attention calculation stage, the text query *Q* interacts element-wise with the visual key *K*, followed by scale normalization and Sigmoid activation to generate channel-aware spatial modulation weights modulated by textual semantics:

(23) $$A=\sigma \left(\frac{Q⊙K}{\sqrt{d}}\right)$$

Here, ⊙ denotes element-wise multiplication, *σ*(∙) denotes the Sigmoid activation function, and *d* represents the attention dimension. This attention weight characterizes the correlation between spatial visual responses and image-level disease semantics, enabling the model to softly enhance semantically consistent small-lesion regions. The dimensional scaling is motivated by the normalization principle used in scaled attention, while projecting query and key representations into a compact latent space can reduce the computational cost of attention modeling [57,58]. In the present n-scale configuration, the P3 feature contains 64 channels; therefore, *d* = 16 reduces the query–key interaction space to one quarter of the original P3 channel dimension while retaining a non-trivial semantic interaction space.

Furthermore, the attention weights are applied to the fused value features, and a 1 × 1 convolution is used to generate the residual enhancement term:

Δ*F* = *Conv*_1×1_(*A*⊙*V*) (24)

Finally, RCMA outputs the enhanced features in a constrained residual form:

*F_out_* = *F* + *α*Δ*F* (25)

Here, *α* is a constrained learnable residual coefficient, which is calculated as follows:

(26) $$\alpha =s\cdot \alpha_{\max}\cdot \mathrm{sigmoid}\left(\alpha_{\log it}\right)$$

Here, ***s*** denotes the runtime scaling coefficient, $\alpha_{\max}$ denotes the upper bound of residual enhancement intensity, and ***α***_log*it*_ is a learnable parameter. Small learnable scaling factors have been used in residual-gating designs to introduce new transformations without abruptly perturbing the original representation [59,60,61]. Following this design principle, RCMA adopts a constrained residual coefficient with *α*_init_ = 0.001 and *α*_max_ = 0.02, so that the cross-modal branch acts as a weak residual correction to the original P3 feature. This constraint is intended to preserve localization-sensitive spatial information while preventing excessive cross-modal modulation.

In summary, RCMA performs cross-modal recalibration of P3/8 small-object features through textual semantic queries, visual key–value matching, high-frequency residual enhancement, and constrained residual injection. This module uses textual semantics to provide image-level disease priors and combines high-frequency residual information to enhance weak boundary and fine-grained texture responses, thereby providing semantically constrained complementary features for small-lesion discrimination, weak-boundary perception, and localization.

### 4.6. PGDAL

Early-stage lesions on potato leaves are usually characterized by small scales, large numbers, weak boundaries, and scattered distributions, where even slight spatial deviations may lead to inaccurate bounding box localization. In particular, when lesions are close to each other, overlapped, or connected, the model may have difficulty establishing a stable correspondence between lesion regions and spatial locations in the early training stage, leading to prediction box shifts, loose bounding boxes, or partial missed detections [62,63,64]. To enhance early spatial learning for localization-sensitive lesion targets, this study introduces PGDAL, which provides additional positional distribution supervision for selected lesion targets at an intermediate feature layer during early training. This module does not modify the original detection head or the main box, cls, and DFL losses and is introduced only as an auxiliary positional supervision term during training.

PGDAL operates on the P4 feature layer. Compared with P3, P4 has stronger semantic representation capability, while retaining higher spatial resolution than P5, making it suitable as an intermediate layer for auxiliary lesion-position supervision [65,66].

Given the P4 feature $F_{p4}\in R^{B\times C\times H_{4}\times W_{4}}$ a lightweight PGDAL head predicts a single-channel positional response map:

***M****_pred_* = *σ*(***h****_pgd_*(***F****_p_*_4_)) (27)

Here, ***h****_pgd_* denotes the PGDAL Head, which consists of a 3 × 3 convolution, BatchNorm, SiLU activation, and a 1 × 1 convolution; *σ*(⋅) denotes the Sigmoid activation function [67,68,69]. This prediction map is used to represent lesion location responses at the P4 scale.

On target, the normalized bounding box of the *j*-th selected lesion is represented as:

*b_j_* = (*c_x_*_,*j*_, *c_y_*_,*j*_, *w_j_*, *h_j_*) (28)

where all four components are normalized to [0, 1]. Given a P4 feature map of size *H*_4_ × *W*_4_, the box is mapped to the P4 coordinate system as:

(29) $$c_{x,j}^{4}=c_{x,j}W_{4},c_{y,j}^{4}=c_{y,j}H_{4},w_{j}^{4}=w_{j}W_{4},h_{j}^{4}=h_{j}H_{4}$$

The horizontal and vertical Gaussian scales are adaptively determined according to the mapped box width and height:

(30) $$\sigma_{x,j}=\max\left(\frac{w_{j}^{4}}{r},\sigma_{\min}\right),\sigma_{y,j}=\max\left(\frac{h_{j}^{4}}{r},\sigma_{\min}\right)$$

Size-adaptive Gaussian heatmaps have been widely used in center- and keypoint-based detectors to provide spatially smooth supervision around annotated object locations [70,71,72]. PGDAL follows this principle by adapting *σ_x_* and *σ_y_* to the mapped lesion width and height. With *r* = 4, the half-width and half-height of a bounding box correspond to approximately 2*σ_x_* and 2*σ_y_*, respectively, when the minimum-scale constraint is inactive. This produces a compact lesion-centered response while retaining smooth supervision over the surrounding spatial region. The lower bound *σ*_min_ = 1 further prevents very small lesions from producing an excessively sharp, nearly single-point target. For the *j*-th selected lesion, the Gaussian response at any spatial location (*x*, *y*) on the P4 feature map is defined as:

(31) $$G_{j}\left(x,y\right)=\exp\left[-\left(\frac{{\left(x-c_{x,j}^{4}\right)}^{2}}{2\sigma_{x,j}^{2}}+\frac{{\left(y-c_{y,j}^{4}\right)}^{2}}{2\sigma_{y,j}^{2}}\right)\right]$$

When multiple lesion targets exist in the same image, their Gaussian responses are fused using the maximum value on the corresponding supervision map, yielding the final Gaussian position supervision map *M_gt_*. This strategy preserves the strongest positional response of different lesion regions while preventing overly strong supervision in dense areas caused by response accumulation.

During target selection, PGDAL adopts a mini-batch-level small-EB-prioritized strategy. For each mini-batch, lesions satisfying w × h ≤ 0.015 and belonging to the EB category are selected first. If no small EB lesion is present in the mini-batch, the target set is relaxed to all EB lesions; if no EB lesion is present, all annotated lesions in the mini-batch are used as a fallback. After target selection, the retained bounding boxes are assigned to their corresponding images according to their batch indices, and a separate P4 Gaussian supervision map is constructed for each image. Therefore, target selection is performed at the mini-batch level, whereas Gaussian target generation is performed at the image level. If an image contains no bounding box retained by the mini-batch-level selection, its Gaussian supervision map remains all zeros.

This priority is intended to emphasize localization-sensitive targets during early training. Small lesions occupy fewer pixels and are more sensitive to spatial deviations; therefore, small EB lesions are assigned the highest priority in the auxiliary target-selection procedure, while the fallback mechanism keeps the auxiliary supervision available for mini-batches without small EB samples.

PGDAL adopts a weighted mean squared error to constrain the consistency between the predicted position map and the Gaussian target map:

(32) $$L_{\mathrm{PGD}}=\mathrm{mean}\left(W\cdot {\left(M_{\mathrm{pred}}-M_{\mathrm{gt}}\right)}^{2}\right)$$

Reweighting foreground and background locations is commonly used in dense detection to prevent the large number of low-information background locations from dominating optimization [70]. In the Gaussian target used by PGDAL, *M*_gt_ > 0.3 corresponds to the central high-response neighborhood within approximately 1.55 Gaussian standard deviations in normalized distance. Locations in this region were therefore assigned a weight of 10.0, whereas the remaining locations retained a nonzero weight of 0.1. This weighting provides stronger supervision around the lesion center while limiting the contribution of the much larger low-response region.

PGDAL is designed as an early-training auxiliary constraint rather than a persistent optimization objective. During the initial training stage, the correspondence between lesion features and spatial locations has not yet been fully established; therefore, Gaussian position-distribution supervision is introduced to provide an additional spatial prior. As training progresses, the primary detection losses gradually provide sufficient localization supervision. To avoid maintaining an additional positional constraint throughout later optimization, PGDAL adopts a warmup-only scheduling strategy. Let *e* denote the zero-based epoch index; the PGDAL weight is defined as follows:

(33) $$\lambda_{\mathrm{pgd}}^{\mathrm{eff}}(e)=\left\{\begin{array}{cc} 0.03, & e<3\\ 0, & e\geq 3 \end{array}\right.$$

That is, PGDAL is enabled only when the epoch index is 0, 1, or 2, and the auxiliary constraint is then disabled. This strategy enables the model to acquire early positional priors for the selected lesion targets and gradually return to the main detection objective in subsequent training.

During TACIM training, the clean branch and the counterfactual branch share the same bounding box annotations; therefore, they use the same Gaussian position supervision map generated from the annotations and compute PGDAL separately during the warmup stage. The final training objective can be written as:

(34) $$L_{\mathrm{total}}=\frac{1}{2}\left(L_{\det}^{\mathrm{clean}}+L_{\det}^{\mathrm{cf}}\right)+\lambda_{\mathrm{feat}}L_{\mathrm{feat}}$$

where, when $\lambda_{\mathrm{pgd}}^{\mathrm{eff}}>0$, we have the following:

(35) $$L_{\det}^{\mathrm{clean}}=L_{\mathrm{yolo}}^{\mathrm{clean}}+\lambda_{\mathrm{pgd}}^{\mathrm{eff}}L_{\mathrm{PGD}}^{\mathrm{clean}}$$

(36) $$L_{\det}^{\mathrm{cf}}=L_{\mathrm{yolo}}^{\mathrm{cf}}+\lambda_{\mathrm{pgd}}^{\mathrm{eff}}L_{\mathrm{PGD}}^{\mathrm{cf}}$$

In summary, PGDAL serves as an auxiliary positional distribution supervision strategy during training, guiding the model to strengthen spatial response learning around the centers and neighboring regions of selected lesion targets during the early training stage without changing the inference pathway. During inference, the model does not invoke the PGDAL head, generate Gaussian position supervision maps, or compute *L*_PGD_; therefore, PGDAL introduces no additional computational overhead during inference. This strategy provides complementary constraints for stable localization of lesions with weak boundaries, dense distributions, and high missed-detection risk.

### 4.7. Experimental Environment and Training Details

To ensure fair and reproducible comparisons, all models were trained and evaluated using the same data splits, hardware, software, and training settings. Experiments were conducted on an NVIDIA GeForce RTX 4090 GPU and an Intel Xeon Platinum 8352V processor under Ubuntu 20.04.5 LTS, Python 3.8.10, PyTorch 2.0.0+cu118, and CUDA 11.8. For the repeated ablation experiments, each configuration was independently trained using the same five prespecified random seeds with deterministic computation enabled. The backbone was initialized with COCO-pretrained YOLO26n weights, whereas the added modules were randomly initialized. Mosaic, HSV perturbation, random translation, random scaling, and horizontal flipping were applied only to the training set. The validation and test sets used deterministic preprocessing. Detailed settings are listed in Table 19. Each model was evaluated on the validation set after every epoch, and the checkpoint with the highest validation fitness was retained. After the architecture and hyperparameters were fixed, the final model was evaluated on the independent test set, which was not used for training, tuning, or model selection. The module-specific numerical values described above were fixed for the present model configuration and kept unchanged across the corresponding comparison experiments. They should be regarded as implementation settings rather than universally optimal constants.

### 4.8. Evaluation Indicators

The detection performance of the proposed model was evaluated using Precision, Recall, F1 Score, mean average precision at an Intersection over Union threshold of 0.5 (mAP50), and mAP50–95. In addition, the number of model parameters and GFLOPs were reported to evaluate model complexity and computational cost.

Average Precision (AP) represents the area under the precision–recall curve for a specific category. The precision–recall curve is generated by varying the confidence threshold and describes the relationship between Precision and Recall at different confidence levels. For category c, AP is calculated as:

(37) $$AP_{c}=\int_{0}^{1}P_{c}\left(R\right)\mathrm{dR}$$

where *P_c_*(*R*) denotes the Precision of category c at a given Recall level.

The mAP50 metric is obtained by averaging the AP values of all lesion categories at an IoU threshold of 0.5:

(38) $$\mathrm{mAP}50=\frac{1}{C}\sum_{c=1}^{C}AP_{c}^{\mathrm{IoU}=0.5}$$

where *C* denotes the number of lesion categories, and $AP_{c}^{\mathrm{IoU}=0.5}$ denotes the AP of category *c* evaluated at an IoU threshold of 0.5. A higher mAP50 value indicates better overall classification and localization performance.

To further evaluate localization accuracy under stricter IoU thresholds, mAP50–95 was also reported. It is calculated by averaging AP over IoU thresholds from 0.50 to 0.95 with a step size of 0.05:

(39) $$\mathrm{mAP}50–95=\frac{1}{10C}\sum_{t\in \left\{0.50,0.55,\ldots ,0.95\right\}}\sum_{c=1}^{C}AP_{c,t}$$

where *AP_c_*_,*t*_ denotes the AP of category *c* evaluated at the IoU threshold *t*. Compared with mAP50, mAP50–95 provides a stricter and more comprehensive evaluation of bounding-box localization accuracy.

To characterize scale-dependent detection difficulty that may be obscured by aggregate performance metrics, lesions were further stratified into small, medium, and large groups according to normalized bounding-box area. AP50 and AP50–95 were then evaluated separately within each lesion-size range. This scale-specific analysis was used to examine whether detection and localization performance varied with lesion size, particularly for small lesions that accounted for a large proportion of the validation annotations.

Precision measures the proportion of correctly detected lesion instances among all instances predicted as lesions:

(40) $$P=\frac{\mathrm{TP}}{\mathrm{TP}+\mathrm{FP}}$$

Recall measures the proportion of ground-truth lesion instances successfully detected by the model:

(41) $$R=\frac{\mathrm{TP}}{\mathrm{TP}+\mathrm{FN}}$$

where *TP* denotes the number of ground-truth lesion instances correctly detected by the model, *FP* denotes the number of background regions, non-target regions, or incorrectly classified objects mistakenly detected as lesions, and *FN* denotes the number of ground-truth lesion instances missed by the model.

The F1 Score is the harmonic mean of Precision and Recall and provides a balanced evaluation of false detections and missed detections:

(42) $$F1=\frac{2\mathrm{PR}}{P+R}$$

The Intersection over Union (IoU) measures the overlap between a predicted bounding box and its matched ground-truth bounding box:

(43) $$\mathrm{IoU}=\frac{\left|B_{\mathrm{pred}}\cap B_{\mathrm{gt}}\right|}{\left|B_{\mathrm{pred}}\cup B_{\mathrm{gt}}\right|}$$

where *B*_pred_ and *B*_gt_ denote the predicted bounding box and the ground-truth bounding box, respectively. A prediction is regarded as a true positive when the predicted category is correct and the IoU between the predicted box and the matched ground-truth box reaches the specified threshold. An unmatched or incorrectly classified prediction is counted as a false positive, whereas an unmatched ground-truth lesion is counted as a false negative.

Precision, Recall, and F1 score were reported at the confidence threshold corresponding to the maximum of the smoothed mean F1 curve across all classes, rather than at a fixed confidence threshold. During validation, predictions were initially filtered using a confidence threshold of 0.001, with an NMS IoU threshold of 0.70 and a maximum of 300 detections per image. mAP50 and mAP50–95 were calculated from the full precision–recall curves.

### 4.9. Statistical Analysis

Statistical analysis was conducted for the module ablation experiments. For the repeated module ablation experiments, each configuration was independently trained using the same five prespecified random seeds under otherwise identical training conditions. Precision, Recall, F1 score, mAP50, and mAP50–95 were summarized as the mean ± standard deviation to describe the average detection performance and variability across repeated runs. To further characterize uncertainty across repeated runs, two-sided 95% confidence intervals for the mean mAP50 and mAP50–95 were calculated using Student’s t distribution with four degrees of freedom and are reported in Table 11.

Statistical analysis was restricted to the repeated module ablation experiments. The low-light experiment was treated as a controlled perturbation-response analysis rather than a repeated statistical experiment. The same trained models were evaluated on six brightness conditions (S0–S5) generated from the same validation images; therefore, these illumination levels were not treated as independent replicates. The resulting performance trend was interpreted descriptively. The text-input ablation, detector-comparison, tomato transferability, and final independent-test experiments were also interpreted descriptively because repeated training results were not available for all corresponding conditions. No claims of statistical significance or seed-independent effect size were made from these single-run experiments.

## 5. Conclusions

To address the challenges posed by small lesion size, low contrast, irregular boundaries, illumination variation, and interference from similar leaf textures, this study proposed a text-guided dual-modal detection model named TRP-Net. The model integrates text-guided semantic information, training-time consistency constraints under environmental perturbations, boundary feature enhancement, and position-aware auxiliary supervision. Together, these components form a unified framework for detecting visible lesions associated with potato early blight and late blight.

On the independent test set, TRP-Net achieved a precision of 78.3%, a recall of 73.7%, an F1 score of 75.9%, an mAP50 of 80.5%, and an mAP50–95 of 34.6%. The detector-side network contained 2.79 M parameters and required 5.2 GFLOPs. In the five-seed controlled ablation experiments on the validation set, the TextGate-only configuration achieved an mAP50 of 75.18 ± 0.48%, compared with 74.66 ± 0.60% for the visual-only Basic model. With TACIM, RCMA, and PGDAL incorporated on top of the shared TextGate pathway, the complete configuration reached an mAP50 of 79.52 ± 0.83% and an mAP50–95 of 34.26 ± 0.25%. These results show that the task-specific components provided additional numerical gains under the common multimodal pathway, while the relatively lower mAP50–95 indicates that precise bounding-box localization under stricter IoU criteria remains an important limitation. The current method is intended for visible-symptom screening and suspected-lesion localization, with final disease confirmation requiring professional assessment or laboratory examination. End-to-end real-time deployment of the complete multimodal pipeline remains to be evaluated in future work.

## Figures and Tables

**Table 1:** Performance comparison under different low-illumination disturbance levels.

Severity	Factor	Basic mAP50	Basic + TACIM mAP50
S0	1.00	75.84	76.62
S1	0.85	75.17	76.01
S2	0.75	74.74	75.75
S3	0.65	73.97	75.38
S4	0.55	72.54	74.68
S5	0.45	69.08	74.21

Note: Basic denotes the original YOLO26n baseline; TACIM, Text-Anchored Counterfactual Intervention Module; mAP50, mean average precision at an intersection-over-union threshold of 0.50. All mAP50 values are reported as percentages. The values in this table were obtained using fixed single checkpoints, whereas the five-seed evaluation results are reported separately in the ablation experiment; therefore, the S0 value should not be directly compared with the corresponding five-seed mean.

**Table 2:** Performance comparison of different feature-enhancement modules.

Models	Precision	Recall	F1	mAP50	mAP50–95	Params (M)
GSPN	73.36 ± 0.33	69.21 ± 0.69	71.22 ± 0.41	76.21 ± 0.91	33.62 ± 0.43	2.77
ODA	72.60 ± 1.71	69.89 ± 0.99	71.21 ± 0.72	76.97 ± 0.78	33.62 ± 0.16	2.94
FDAM	74.37 ± 1.12	70.32 ± 1.96	72.27 ± 0.97	76.60 ± 0.60	33.03 ± 0.24	2.77
RCMA	75.61 ± 1.32	72.43 ± 0.80	73.99 ± 0.82	77.94 ± 0.90	33.55 ± 0.69	2.79

Note: F1, F1 score; mAP50, mean average precision at an intersection-over-union threshold of 0.50; mAP50–95, mean average precision averaged over intersection-over-union thresholds from 0.50 to 0.95; Params, number of model parameters; M, million; GSPN, Generalized Spatial Propagation Network; ODA, Object-Difference Attention; FDAM, Frequency-Dynamic Attention Modulation; RCMA, Residual Cross-Modal Attention. Performance metrics are reported as the mean ± standard deviation across five random seeds.

**Table 3:** Performance of PGDAL.

Models	Precision	Recall	F1	mAP50	mAP50–95
MALoss	74.28 ± 1.11	68.45 ± 1.60	71.23 ± 0.54	75.71 ± 0.70	32.70 ± 0.36
Varifocal	74.25 ± 1.97	67.12 ± 1.46	70.48 ± 1.17	71.30 ± 0.76	30.70 ± 0.46
PGDAL	74.94 ± 0.98	69.10 ± 1.64	71.90 ± 1.31	76.33 ± 1.29	33.94 ± 0.49

Note: F1, F1 score; mAP50, mean average precision at an intersection-over-union threshold of 0.50; mAP50–95, mean average precision averaged over intersection-over-union thresholds from 0.50 to 0.95; MALoss, Multiple Anchor Learning loss; Varifocal Loss, an IoU-aware classification loss; PGDAL, P4 Gaussian Position Distribution Auxiliary Loss. Performance metrics are reported as the mean ± standard deviation across five random seeds.

**Table 4:** Ablation experiment of TRP-Net.

Models	P	R	F1	mAP50	mAP50–95	Params (M)
basic	72.28 ± 1.60	68.33 ± 0.91	70.23 ± 0.41	74.66 ± 0.60	32.10 ± 0.39	2.38
TextGate	72.76 ± 1.78	71.32 ± 1.61	72.01 ± 0.65	75.18 ± 0.48	31.32 ± 0.79	2.78
TACIM	75.36 ± 0.96	69.74 ± 0.75	72.43 ± 0.53	76.73 ± 0.90	34.15 ± 0.24	2.78
RCMA	75.61 ± 1.32	72.43 ± 0.80	73.99 ± 0.82	77.94 ± 0.90	33.55 ± 0.69	2.79
PGDAL	74.94 ± 0.98	69.10 ± 1.64	71.90 ± 1.31	76.33 ± 1.29	33.94 ± 0.49	2.78
TACIM + RCMA	75.52 ± 1.25	72.70 ± 1.28	74.07 ± 0.74	78.93 ± 0.57	34.14 ± 0.19	2.79
TACIM + PGDAL	74.65 ± 1.35	72.13 ± 1.07	73.36 ± 0.56	77.99 ± 0.69	33.54 ± 0.16	2.78
RCMA + PGDAL	76.03 ± 1.31	71.92 ± 1.06	73.91 ± 0.72	78.84 ± 0.82	33.70 ± 0.31	2.79
TACIM + RCMA + PGDAL	77.10 ± 1.51	73.25 ± 1.20	75.11 ± 0.86	79.52 ± 0.83	34.26 ± 0.25	2.79

Note: P, Precision; R, Recall; F1, F1 score; mAP50, mean average precision at IoU = 0.50; mAP50–95, mean average precision over IoU thresholds from 0.50 to 0.95; Params, number of inference-time parameters; M, million. TACIM, Text-Anchored Counterfactual Intervention Module; RCMA, Residual Cross-Modal Attention; PGDAL, P4 Gaussian Position Distribution Auxiliary Loss. Basic denotes the visual-only YOLO26n baseline, whereas all other configurations share the TextGate image–text fusion pathway; thus, their parameter counts include TextGate. TACIM and PGDAL are training-time components and introduce no additional inference-time branches. Results are reported as mean ± standard deviation over five random seeds. All configurations require approximately 5.2 GFLOPs at an input size of 768 × 768; therefore, GFLOPs are not listed separately.

**Table 5:** 95% confidence intervals for the five-seed controlled ablation experiments.

Models	mAP50 95% CI	mAP50–95 95% CI
basic	73.92–75.40	31.62–32.58
TextGate	74.58–75.78	30.34–32.30
TACIM	75.61–77.85	33.85–34.45
RCMA	76.82–79.06	32.69–34.41
PGDAL	74.73–77.93	33.33–34.55
TACIM + RCMA	78.22–79.64	33.90–34.38
TACIM + PGDAL	77.13–78.85	33.34–33.74
RCMA + PGDAL	77.82–79.86	33.32–34.08
TACIM + RCMA + PGDAL	78.49–80.55	33.95–34.57

Note: Confidence intervals were calculated from the mean and standard deviation of five independently trained runs using Student’s t distribution with four degrees of freedom.

**Table 6:** Performance comparison under different PGDAL warmup durations.

Warmup Epochs	P	R	F1	mAP50	mAP50–95
1	75.33 ± 0.80	72.16 ± 1.03	73.71 ± 0.67	77.75 ± 0.70	33.49 ± 0.22
3	77.10 ± 1.51	73.25 ± 1.20	75.11 ± 0.86	79.52 ± 0.83	34.26 ± 0.25
5	76.35 ± 1.82	70.86 ± 1.34	73.49 ± 1.11	77.96 ± 1.07	33.63 ± 0.22

Note: All results are reported as mean ± standard deviation over five random seeds. Only the duration of PGDAL activation was varied, while the remaining training settings were kept unchanged.

**Table 7:** Text-input ablation results of TRP-Net on the validation set.

Text Setting	P	R	F1	mAP50	mAP50–95	EB AP50	EB AP50–95	LB AP50	LB AP50–95
original	76.93	74.12	75.50	80.36	34.36	73.18	28.45	87.54	40.26
zero	78.30	71.23	74.60	78.24	34.05	72.01	28.45	84.46	39.66
neutral	75.55	74.48	75.01	79.43	34.04	71.84	28.19	87.02	39.89
shuffled	76.53	73.49	74.98	79.76	34.19	72.11	28.33	87.41	40.05
No location	76.22	72.87	74.51	80.14	34.59	73.08	28.48	87.20	40.69

Note: P, Precision; R, Recall; F1, F1 score; AP50, average precision at an intersection-over-union threshold of 0.50; AP50–95, average precision averaged over intersection-over-union thresholds from 0.50 to 0.95; mAP50, mean AP50 across the disease classes; mAP50–95, mean AP50–95 across the disease classes; EB, early blight; LB, late blight. All values are reported as percentages. Each text-input condition was evaluated using a single training run with the same fixed random seed.

**Table 8:** Detection performance of TRP-Net across different disease classes.

Class	AP50	AP50–95
EB	73.18	28.45
LB	87.54	40.26
Mean	80.36	34.36

Note: EB, early blight; LB, late blight; AP50, average precision at an intersection-over-union threshold of 0.50; AP50–95, average precision averaged over intersection-over-union thresholds from 0.50 to 0.95. Mean denotes the arithmetic mean of the AP values for EB and LB. All values are reported as percentages.

**Table 9:** Scale-specific detection performance of the original-text model.

Scale	Number	Proportion	AP50	AP50–95
Small	2513	74.84	60.78	19.40
Medium	535	15.93	64.28	22.68
Large	310	9.23	61.23	28.88

Note: Lesion scale was determined using the normalized bounding-box area *a* = *w* × *h*. Small, medium, and large lesions were defined as *a* ≤ 0.015, 0.015 < *a* < 0.05, and *a* ≥ 0.05, respectively. Number denotes the number of annotated lesion instances in each scale group, and Proportion denotes its percentage among all 3358 lesion instances in the validation set. AP50 and AP50–95 are reported as percentages.

**Table 10:** Performance comparison of TRP-Net and other detectors.

Models	Precision	Recall	F1	mAP50	mAP50–95	Params (M)	GFLOPs
Ours	78.3	73.7	75.9	80.5	34.6	2.79	5.2
YOLO11n	72.8	70.3	71.5	73.3	30.3	2.6	6.5
DEIM	72.1	67.0	69.4	70.7	28.2	3.7	7.1
DETR	55.2	49.3	52.1	43.0	13.9	41.5	86.0
D-FINE	75.3	69.3	72.2	73.7	30.1	4.7	7.1
Faster R-CNN	69.7	70.0	69.8	70.8	26.6	41.3	133.9
RetinaNet	69.0	68.0	68.5	67.3	24.9	32.2	126.9
RT-DETRv2	73.4	68.2	70.7	73.8	29.4	36.0	120.0
SSD	68.8	67.2	68.0	66.9	23.3	23.8	30.4

Note: F1, F1 score; mAP50, mean average precision at an intersection-over-union threshold of 0.50; mAP50–95, mean average precision averaged over intersection-over-union thresholds from 0.50 to 0.95; Params, number of model parameters; M, million; GFLOPs, giga floating-point operations. Precision, Recall, F1, mAP50, and mAP50–95 are reported as percentages.

**Table 11:** Detection performance of TRP-Net on the full test set and natural-scene subset.

Test Subset	Precision	Recall	F1	mAP50	mAP50–95
Full test	78.3	73.7	75.9	80.5	34.6
Natural-scene	75.2	72.9	74.0	79.1	33.7

**Table 12:** Composition of the tomato leaf disease dataset used in the architecture-transferability experiment.

Split	Images	EB Lesions	LB Lesions	Total Lesions
Training	1551	2160	1215	3375
Validation	539	818	549	1367
Test	562	1554	800	2354
Total	2652	4532	2564	7096

**Table 14:** Public potato leaf image datasets used in this study.

Datasets	Original Images	Link
PlantVillage	2152	https://github.com/spMohanty/PlantVillage-Dataset (accessed on 10 September 2026)
Potato Disease Leaf Dataset (PLD)	4062	https://www.kaggle.com/datasets/rizwan123456789/potato-disease-leaf-datasetpld (accessed on 10 September 2026)
Potato Computer Vision Model	424	https://universe.roboflow.com/safety-v4ef0/potato-e1q5p (accessed on 10 September 2026)
Healthy Leaf Dataset	98	https://universe.roboflow.com/project-urge/healthy-leaf-g4tgq (accessed on 10 September 2026)

**Table 15:** Distribution of retained potato leaf images by source and data split.

Datasets	Train	Val	Test	Total
PlantVillage	1300	280	0	1580
Potato Disease Leaf Dataset (PLD)	800	174	0	974
Potato Computer Vision Model	0	0	365	365
Healthy Leaf Dataset	0	0	85	85

**Table 16:** Dataset composition and distribution across data splits.

Data Split	EB Images	LB Images	Healthy Images	EB Boxes	LB Boxes	Total Images	Total Boxes
Train	1000	647	453	8417	1965	2100	10,382
Val	201	200	53	2882	476	454	3358
Test	90	283	77	1213	849	450	2062
Total	1291	1130	583	12,512	3290	3004	15,802

**Table 17:** Distribution of controlled-background and natural-scene images across dataset splits.

Data Split	Controlled- Background	Natural-Scene	Total
Train	265	1835	2100
Val	92	362	454
Test	78	372	450

**Table 18:** Representative examples of manually corrected image descriptions from potato late blight images.

Image Identifier	Original LLaVA Description	Corrected Description
7.jpg	A large number of small brown irregular lesions scattered everywhere	A large number of large brown irregular lesions scattered on one side of leaves
231.jpg	Numerous large brown irregular lesions, unevenly distributed	A few small brown irregular lesions, unevenly distributed
294.jpg	Numerous small brown irregular lesions, unevenly distributed	Numerous small brown irregular lesions mainly distributed on leaf tips

The examples were selected from manually corrected cases among potato late blight images with identifiers 1–500 and are presented to illustrate the manual review and correction procedure.

**Table 19:** Experimental environment and training configurations.

Environment	Items	Configuration
Hardware environment	GPUs	NVIDIA RTX 4090 GPU (NVIDIA Corporation, Santa Clara, CA, USA)
	CPU	Intel Xeon Platinum 8352V
Software environment	PyTorch	PyTorch 2.0 (Meta AI, New York, NY, USA)
	Python	3.8.10
	Ubuntu	20.04.5 LTS
	CUDA	11.8
Training settings	Optimizer	SGD
	Initial learning rate	0.01
	Batch size	16
	Epochs	200
	Input image size	768 × 768
	Data augmentation	Mosaic1.0;HSV0.015/0.7/0.4; translation0.1;scaling0.5; horizontal flipping 0.5
	Disabled augmentation	MixUp, vertical flipping, rotation, shear and perspective
Visualization	Confidence threshold	0.25
Model selection	Criterion	Highest validation fitness

## Data Availability

The source code for implementing TRP-Net is publicly available at GitHub: https://github.com/chaojiggggood-gif/TRPNet (accessed on 10 September 2026).
